# Silicon clusters with six and seven unsubstituted vertices *via* a two-step reaction from elemental silicon†
†Dedicated to Professor Reinhold Tacke on the occasion of his 70^th^ birthday.
‡
‡Electronic supplementary information (ESI) available. CCDC 1896557 and 1896556. For ESI and crystallographic data in CIF or other electronic format see DOI: 10.1039/c9sc03324f


**DOI:** 10.1039/c9sc03324f

**Published:** 2019-08-15

**Authors:** Lorenz J. Schiegerl, Antti J. Karttunen, Wilhelm Klein, Thomas F. Fässler

**Affiliations:** a Department of Chemistry , Technische Universität München , Lichtenbergstraße 4 , 85748 Garching , Germany . Email: thomas.faessler@lrz.tum.de; b WACKER Institute of Silicon Chemistry , Technische Universität München , Lichtenbergstraße 4 , 85748 Garching , Germany; c Department of Chemistry and Materials Science , Aalto University , 00076 Aalto , Finland

## Abstract

Unsaturated silicon clusters with only partial substitution, and thus, “naked” Si atoms are well studied species as they are proposed intermediates in gas-phase deposition processes. Although a remarkable number of stable molecular clusters has been reported, they are typically still obtained by multi-step syntheses. Herein we introduce a newly developed synthetic approach which led to the formation of the anionic species {Si(TMS)_3_}_3_Si_9_^–^ (**1a**) and {Si(TMS)_3_}_2_Si_9_^2–^ (**1b**), and an extension of this synthetic protocol resulted in the first covalent attachment of ligands through metal atoms to these clusters, (SnCy_3_)_3_Si_9_^–^ (**2a**) and (SnCy_3_)_2_Si_9_^2–^ (**2b**). The influence of the substituents on the electron localization in the central Si_9_ unit is analyzed by means of intrinsic bond orbital (IBO) analysis and partial atomic charge distribution. The IBO analyses reveal a new type of delocalization including 5-center-6-electron besides 3-center-2-electron bonds. The Raman spectra of **1b** and **2b** allow an assignment of the Si–Si intra-cluster vibrations by comparison to calculated (DFT-PBE0) spectra. The anions are formed in a one-step synthesis from binary K_12_Si_17_ which can easily be obtained by fusing the elements K and Si. The anions are characterized by ESI mass spectrometry and comprehensive NMR studies (^1^H, ^13^C, ^29^Si, ^119^Sn). Attempts to crystallize **1a** and **2a** as their (K–222crypt)^+^ salts yielded after the loss of one of the substituents single crystals containing **1b** and **2b**. The single crystal X-ray structure analyses reveal the presence of anionic siliconoids with surfaces of seven unsubstituted silicon atoms.

## Abstract 2

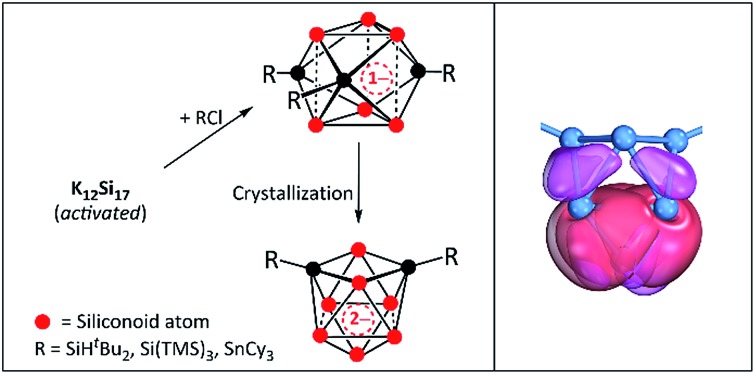
A synthetic shortcut to molecules that contain several unprotected silicon atoms comprising the whole range from localized to delocalized Si–Si bonds.

## Introduction

**Fig. 1:**
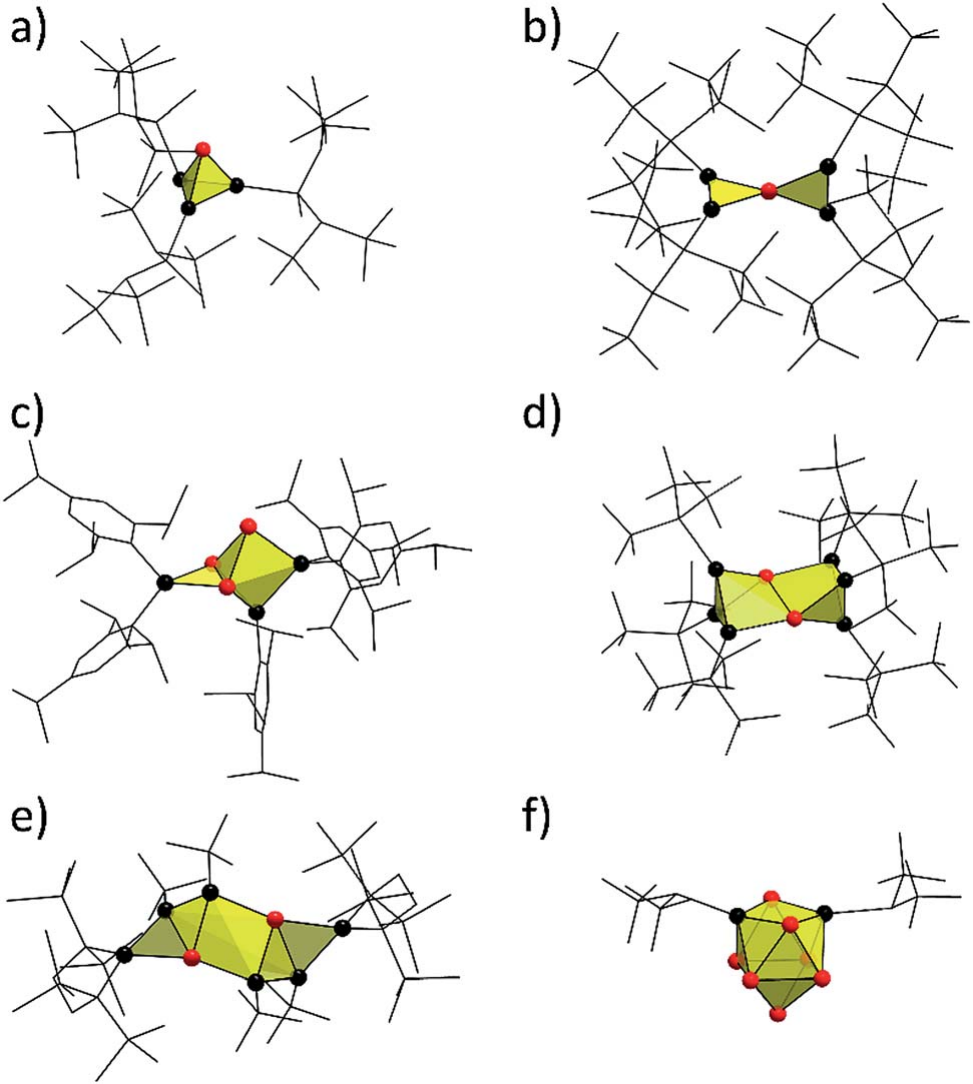
Selected examples of known molecular silicon cluster species with naked cluster atoms: (a) (SiMeDis_2_)_3_Si_4_^–^ (Dis = CH(SiMe_3_)_2_);^28^ (b) {Si(SiMe^*t*^Bu_2_)_3_}_4_Si_5_;^29^ (c) the siliconoid (Tip)_5_Si_6_^–^ (Tip = 2,4,6-triisopropylphenyl);^18^ (d) (Si^*t*^Bu_3_)_6_Si_8_;^21^ (e) (^*t*^Bu)_4_{C_4_(SiMe_3_)_4_}_2_Si_8_;^20^ (f) the siliconoid (SiH^*t*^Bu_2_)_2_Si_9_^2–^ (**3b**).^30^ Silicon clusters are shown as yellow polyhedra; naked and substituted Si cluster atoms are shown as red and black spheres, respectively, and the cluster substituents are drawn in the wire-and-stick mode.

The call for new sources of silicon-based materials is steadily increasing due to applications in numerous daily-life products as *e.g.* batteries, photovoltaics and electronic devices.^1^–^8^ The wide range of applications is promoted by the abundancy, low costs, non-toxicity and semiconducting properties of silicon. After the first reports on Si=Si double bonds^9^,^10^ a new field for the exploration of tailor-made low-valent silicon compounds has been established that undergoes constant expansion, mirrored *e.g.* by the synthesis of stable silaethenes (SiMe_3_)_2_Si=C(OSiMe_3_)R (R = adamantyl, CEt_3_, CMe),^11^,^12^ compounds with silicon–silicon triple bonds as in (^i^PrR′_2_)Si–Si≡Si–Si(R′_2_^i^Pr) (R′ = CH(SiMe_3_)_2_),^13^ a stable silylene (CH)_2_(NC(CH_3_)_3_)_2_Si,^14^ an aromatic hexasilabenzene isomer (Tip)_6_Si_6_ (Tip = 2,4,6-triisopropylphenyl),^15^ the triatomic Si(0) unit (CAAC)_3_Si_3_ (CAAC = cyclic (alkyl)amino carbene),^16^ and of so-called siliconoid clusters.^17^–^21^ Siliconoids are best described as partially substituted silicon clusters with ligand-free silicon atoms (Fig. 1), and further modifications of such silicon compounds have frequently been achieved, which underlines their versatile synthetic potential for the formation of silicon-based materials.^19^–^27^

Their synthesis has made major progress in recent years, and just lately a step-wise, atomically precise expansion of the anionic siliconoid (Tip)_5_Si_6_^–^ (Fig. 1b) was reported using (Cp*)_2_Si.^31^ The formation of siliconoids of higher nuclearity from molecular precursors, however, generally affords several synthetic steps. In 1993, Wiberg *et al.* already suggested in their report on the synthesis of (Si^*t*^Bu_3_)_4_Si_4_ that such substituted Si_4_ cluster compounds should probably be accessible in a more straightforward manner through the reaction of alkyl halides with tetrahedral Si_4_^4–^ polyanions that occur in binary alkali metal alloys of silicon.^32^ This idea was promoted by subsequent reports on (SiMeDis_2_)_3_Si_4_^–^ (Dis = CH(SiMe_3_)_2_)^28^ (Fig. 1a) and (Si^*t*^Bu_3_)_3_Si_4_^–^ (ref. 33) in which – again *via* molecular precursors – tri-substituted tetrahedral Si_4_ clusters have been obtained. The idea of using *Zintl* anions as precursors for Si-rich molecules has constantly been pursued for more than two decades, nevertheless, many approaches were repeatedly discarded due to the high reducing properties of such silicides.^19^,^32^

Examples of *Zintl* phases with deltahedral clusters are A_4_Si_4_ (ref. 34–37) (A = Li–Cs) and A_12_Si_17_ (ref. 38 and 39) (A = K–Cs), which contain solely Si_4_^4–^ units and Si_4_^4–^ alongside Si_9_^4–^ in a 2 : 1 ratio, respectively. The Si_4_^4–^ and Si_9_^4–^ clusters are unsaturated species with interesting properties firstly due to their nucleophilic character (multiple negative charge). Secondly, they are electrophilic in character at the same time due to an electron-deficient bonding situation of the cluster skeleton.^40^,^41^ Since the A_4_Si_4_ phases are rather insoluble in any solvent, the focus was set on the A_12_Si_17_ phases which are soluble in liquid ammonia, from which solvates containing Si_4_^4–^ (ref. 42 and 43) and Si_9_^4–^ (ref. 43–45) units could be obtained, and it has been shown that the Si_4_^4–^*Zintl* clusters from such an A_12_Si_17_ phase are receptive to chemical conversion. (CuMes)_2_Si_4_^4–^ (ref. 46) was obtained by the reaction with CuMes (Mes = 1,3,5-trimethylbenzene). But also the Si_9_^4–^ clusters could be derivatized *e.g.* by the addition of transition metal fragments yielding (PhZn)Si_9_^3–^,^47^ ({Ni(CO)_2_}_2_Si_9_)_2_^8–^ (ref. 48) and (NHC^Dipp^Cu)Si_9_^3–^ (NHC^Dipp^ = 1,3-bis-(2,6-di-iso-propylphenyl)imidazole-2-ylidene).^49^

However, a synthetic approach including Si_4_^4–^ or Si_9_^4–^ units to form covalent bonds to ligands is still missing, although such reactions of the corresponding Ge_9_^4–^ clusters (from the precursor *Zintl* phase K_4_Ge_9_)^50^–^53^ are very well known. Investigations on the solubility of the silicon clusters in A_12_Si_17_ revealed that in liquid ammonia solution the mono-protonated species HSi_9_^3–^ (ref. 45 and 54) is present, and that a subsequent transfer to pyridine yields even the doubly-protonated species H_2_Si_9_^2–^.^55^ Furthermore, theoretical studies suggest the formation of siliconoids with Si_9_^4–^ units by the attachment of sp^3^-Si linkers.^56^ Just recently, we used Si_9_ clusters from the precursor K_12_Si_17_ for the production of the anionic siliconoids (SiH^*t*^Bu_2_)_3_Si_9_^–^ (**3a**) and (SiH^*t*^Bu_2_)_2_Si_9_^2–^ (**3b**, Fig. 1d) *via* direct ligand attachment.^30^ The silylation of Si_9_ clusters by the reaction of K_12_Si_17_ with SiH^*t*^Bu_2_Cl yields species with covalently bonded SiH^*t*^Bu_2_ groups at the cluster vertex atoms. These siliconoids contain the so far highest number of unsubstituted silicon atoms (six in **3a** and seven in **3b**) and are more easily accessible than those siliconoids obtained *via* the established “molecular multi-step” approaches. By a recent definition of siliconoids,^19^ the unsubstituted Si atoms only display homoatomic bonds with a hemispheroidal coordination sphere and are free of ligands. Herein we report on the reactivity of K_12_Si_17_ towards SiTMS_3_Cl and SnCy_3_Cl (TMS = trimethylsilane, Cy = cyclohexyl).

## Results and discussion

The recently introduced synthetic route for the formation of anionic siliconoids by substitution of Si_9_ clusters from K_12_Si_17_ was further explored employing SiTMS_3_Cl and SnCy_3_Cl as reagents. Reactions of Ge_9_ clusters from the K_4_Ge_9_ precursor with such reactants led to an attachment of silyl^51^,^57^–^61^ and stannyl^62^,^63^ groups at the cluster cores, and the products were characterized as tri-substituted cluster species by ESI-MS and NMR investigations in solution as well as by X-ray analysis in solid-state. Regarding the corresponding reaction of Si_9_ clusters from K_12_Si_17_ in solution, an “*activation*” of the precursor by liquid NH_3_/222crypt^30^,^55^ has been found to be a key-step. The silylation of Si_9_ was accomplished by the reaction of K_12_Si_17_ after pretreatment with liquid NH_3_/222crypt^30^ with Si(TMS)_3_Cl and SnCy_3_Cl in thf and pyridine, respectively, and yielded deep brownish filtrates which were dried *in vacuo*. Digesting the residue with fluorobenzene, decanting of the solutions, and removal of all volatile ingredients yielded bulk materials which were further characterized by ESI-MS and NMR spectroscopy (^1^H, ^13^C, ^119^Sn, ^29^Si).

**Fig. 2:**
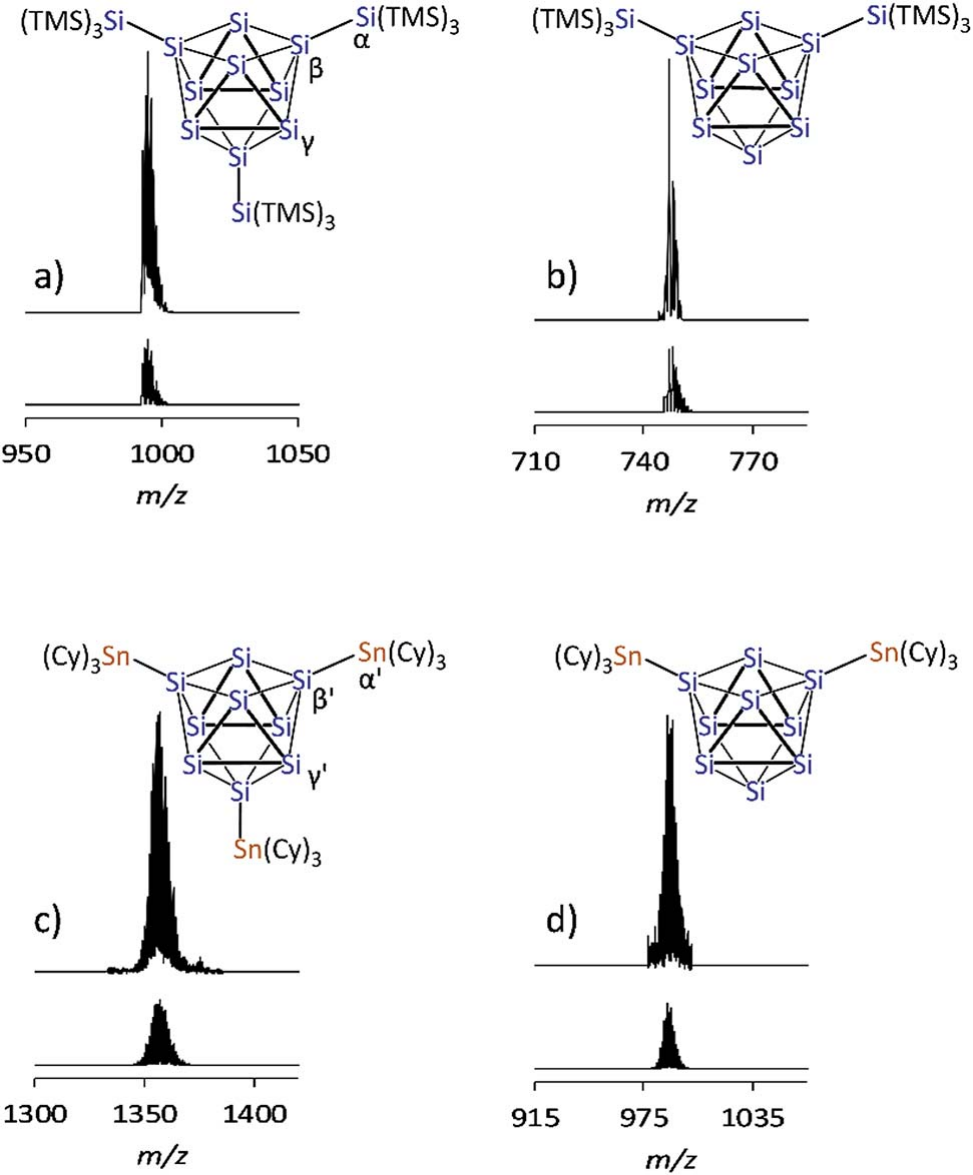
ESI-MS mass peaks of the Si_9_ species (top: measured spectrum, bottom: simulated isotope pattern): (a) {Si(TMS)_3_}_3_Si_9_ (**1a**), *m*/*z* = 996; (b) {Si(TMS)_3_}_2_Si_9_ from mass fragmentation (**1b**), *m*/*z* = 748; (c) (SnCy_3_)_3_Si_9_ (**2a**), *m*/*z* = 1357; (d) (SnCy_3_)_2_Si_9_ (**2b**), *m*/*z* = 989. For details of the measurement, see ESI.‡

The ESI-MS spectrum of the reaction product with SiTMS_3_Cl shows the mass peak of the tri-silylated species {Si(TMS)_3_}_3_Si_9_^–^ (**1a**) as single species (Fig. 2a). Fragmentation of the isolated mass peak leads to the corresponding di- and mono-silylated cluster species (Fig. 2b) and confirms the composition of **1a**. The isotope distribution of the mass peaks comprises a unit of 21 silicon atoms in accordance with the presence of three Si(TMS)_3_ substituents at the Si_9_ cluster. In analogy, the ESI-MS spectrum of the reaction product with SnCy_3_Cl shows the mass peak for the tri-stannylated species (SnCy_3_)_3_Si_9_^–^ (**2a**) (Fig. 2c). However, the mass peak of the di-stannylated species (SnCy_3_)_2_Si_9_^–^ (Fig. 2d) was also present, although with lower intensity. A fragmentation mass experiment of **2a** confirmed the composition by the loss of SnCy_3_ in analogy to **1a** and **3a**.^30^

^1^H and ^13^C NMR spectra of **1a** (NMR spectra in the ESI‡) exclusively show one type of TMS group (^1^H: 0.25 ppm; ^13^C: 3.51 ppm) and one set of signals for 222crypt (^1^H: 3.64, 3.59, 2.61 ppm; ^13^C: 71.47, 67.44, 55.01 ppm). The ^1^H NMR integral ratio of TMS : 222crypt = 3 : 1 confirms the composition of a triply silylated species for **1a** (Fig. 2a). The ^1^H and ^13^C NMR spectra of **2a** show the expected signals for the cyclohexyl groups. The Cy signals appear superimposed due to their signal splitting, but the ^13^C NMR spectrum reveals four peaks (33.96, 31.10, 25.86, 21.54 ppm) for the SnCy_3_ groups of **2a**. A ^119^Sn NMR measurement reveals one single peak at –70.50 ppm, indicative of one sort of SnCy_3_ groups in the reaction product, although the integral ratio (SnCy_3_ : 222crypt) in the ^1^H NMR measurement does not perfectly match a ratio of 3 : 1 of **2a**. Most likely, small amounts of side-products containing (K–222crypt)^+^ units are responsible for this observation.

The ^29^Si NMR spectrum of a solution of the bulk material containing **1a** reveals four signals at –8.70, –129.94, –175.29, and –360.72 ppm, indicative of a tri-silylated *D*_3h_ symmetric Si_9_ core. The signals at –8.70 ppm (TMS) and –129.94 ppm (α, Fig. 2a) originate from the attached silyl groups and conform well to reported shifts of the corresponding Ge_9_ species {Si(TMS)_3_}_3_Ge_9_^–^.^51^,^60^ The signals at –175.29 (β, Fig. 2a) and –360.72 ppm (γ, Fig. 2a) match well with the signals reported for the Si_9_ cluster atoms of (SiH^*t*^Bu_2_)_3_Si_9_^–^ (**3a**) (–175.16 and –358.81 ppm, which were confirmed in computational studies).^30^ The ^29^Si NMR spectrum of **2a** reveals signals at –100.01 (β′, Fig. 2c) and –335.50 ppm (γ′, Fig. 2c). The signals of the substituted cluster atoms β/β′ are shifted more downfield if compared to the one of the ligand-free cluster atoms of the prismatic faces γ/γ′, which bear the highest negative ppm values for known siliconoids.^19^ The shift range of γ/γ′ is comparable to that of the protonated species H_2_Si_9_^2–^ (–346 ppm)^55^ and HSi_9_^3–^ (–359 ppm).^54^

**Fig. 3:**
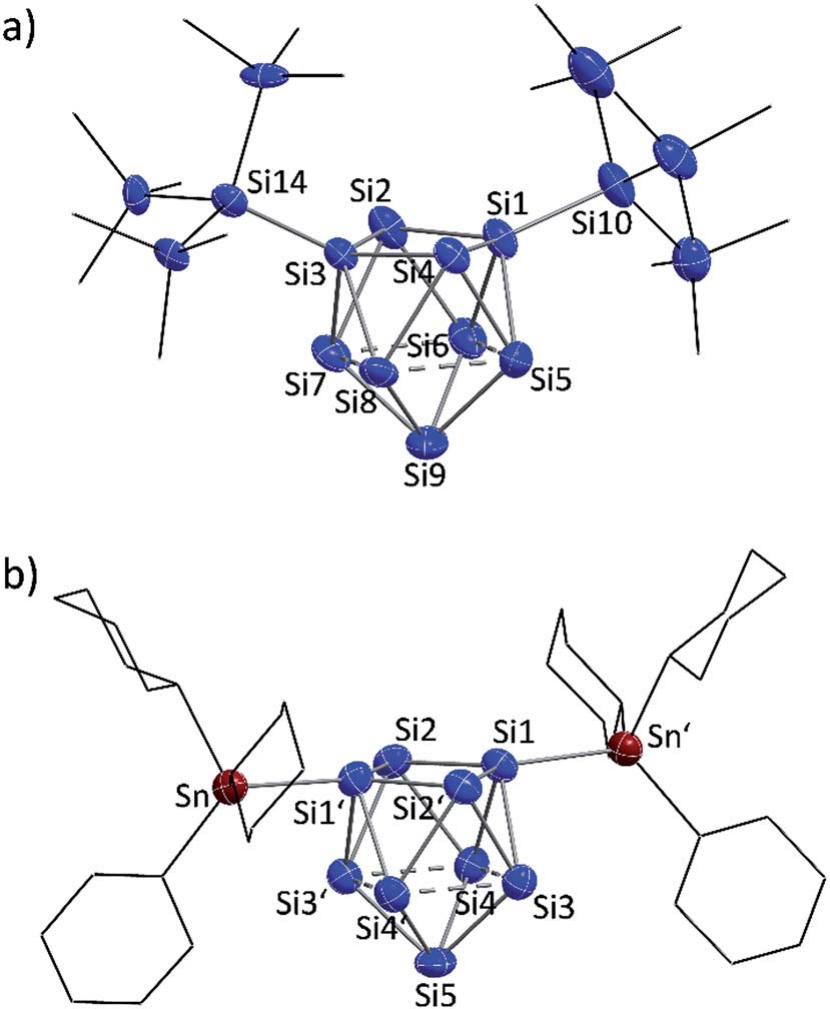
Single crystal structures of di-anionic siliconoids: (a) {Si(TMS)_3_}_2_Si_9_^2–^ (**1b**); (b) (SnCy_3_)_2_Si_9_^2–^ (**2b**); symmetry operation: (′) = –*x*, *y*, 0.5 – *z*; Si and Sn atoms (blue and red-brown, respectively) are shown as ellipsoids at 50% probability level; C atoms are drawn in the wire-and-stick mode; H atoms are omitted.

Yellow block-shaped crystals suitable for single crystal X-ray diffraction were obtained from fluorobenzene/hexane solutions of bulk materials **1a** and **2a**. However, the structure determinations show the presence of the di-anionic siliconoid species {Si(TMS)_3_}_2_Si_9_^2–^ (**1b**) and (SnCy_3_)_2_Si_9_^2–^ (**2b**). The occurrence of the di-substituted species in the single crystals is traced back to substituent cleave from the cluster cores during crystallization. Ligand scrambling has been observed before in anionic Si_4_ clusters.^28^ However, a disproportionation according to “2{Si(TMS)_3_}_3_Si_9_^–^ → {Si(TMS)_3_}_2_Si_9_^2–^ + {Si(TMS)_3_}_4_Si_9_” is excluded by to calculations due to the strongly endoenergetic nature of +92 kJ mol^–1^ (further information in ESI‡). EDX analyses confirmed the corresponding K : Si (**1b**) and K : Si : Sn (**2b**) ratios in the single crystals. The molecular structures of the siliconoid di-anions **1b** and **2b** are shown in Fig. 3a and b, respectively. The structures can be described as di-substituted Si_9_ clusters with the shape of a *C*_2v_-distorted mono-capped square anti-prism. The substituents are attached at two opposing silicon vertex atoms of the open square of the cluster.

Beside the Si–Si and Si–Sn exo-bonds, the nine Si atoms of the central units in **1b** and **2b** display two groups of Si–Si bond lengths, which are listed and compared to the ones in the known anion **3b** in Table 1 (type 1 and type 2). The shorter bonds (type 1) in the range between 2.40 and 2.48 Å are slightly longer than typical single bonds. Interestingly, the elongations are quite small considering that Si1/Si3 in **1a** and Si1/Si1′ in **2b** show a coordination number of 5. A comparison to the bond lengths within the non-capped square in the cluster HSi_9_^3–^ shows a significant influence of the hydrogen substituent on the Si–Si distances. The bonds within the square with the substituted Si atom (coordination number 5) of 2.34 Å are clearly shorter if compared to the ones between the unsubstituted atoms (2.55 Å).^45^,^54^ By contrast, the atoms Si9 (**1a**) and Si5 (**2b**) with coordination number 4 form an umbrella-type coordination to the neighboring Si atoms. Most intriguingly, the atoms Si5 to Si8 in **1b** and Si3 to Si4 in **2b** each show, beside three shorter contacts of type 1, two longer contacts to atoms of the same kind (type 2). The corresponding Si–Si distances between 2.569(6)–2.738(5) Å in **1b** and 2.565(2)–2.664(2) Å in **2b** are in the typical region of unsubstituted Si atoms with an umbrella-type coordination sphere. Distances between such silicon atoms in known siliconoids as *e.g.* (Tip)_5_Si_6_^–^ (2.5506(9) Å, Fig. 1b),^18^ (Tip)_6_Si_6_ (2.7076(8) Å)^22^ and (Mes)_6_Si_5_ (2.636(1) Å)^64^ are comparable to the type 2 bonds.

**Table 1:** Selected interatomic distances [Å] in the molecular structures of the siliconoid anions **1b**, **2b** and **3b** from single crystal structure determinations (molecular structures with atom labelling in Fig. 3 and 4)

Bond type	{Si(TMS)_3_}_2_Si_9_^2–^ (**1b**)	(SiH^*t*^Bu_2_)_2_Si_9_^2–^ (**3b**)^30^	(SnCy_3_)_2_Si_9_^2–^ (**2b**)
Exo-bonds	Si1–Si10: 2.357(5)	Si1–Si10: 2.349(2)	Si1–Sn: 2.578(1)
	Si3–Si14: 2.339(5)	Si3–Si11: 2.379(2)	Si1′–Sn′: 2.578(1)
Type 1	Si1–Si2: 2.396(5)	Si1–Si2: 2.405(2)	Si1–Si2: 2.430(2)
	Si1–Si4: 2.427(5)	Si1–Si4: 2.423(2)	Si1–Si2′: 2.433(2)
	Si2–Si3: 2.395(4)	Si2–Si3: 2.418(2)	Si1′–Si2: 2.433(2)
	Si3–Si4: 2.398(5)	Si3–Si4: 2.414(2)	Si1′–Si2′: 2.430(2)
	Si5–Si9: 2.468(6)	Si5–Si9: 2.429(2)	Si4–Si5: 2.436(2)
	Si6–Si9: 2.470(6)	Si6–Si9: 2.441(2)	Si3–Si5: 2.432(2)
	Si7–Si9: 2.427(6)	Si7–Si9: 2.450(2)	Si4′–Si5: 2.436(2)
	Si8–Si9: 2.475(7)	Si8–Si9: 2.447(2)	Si3′–Si5: 2.432(2)
Type 2	Si5–Si6: 2.772(6)	Si5–Si6: 2.782(2)	Si3–Si4: 2.664(2)
	Si7–Si8: 2.738(5)	Si7–Si8: 2.764(2)	Si3′–Si4′: 2.664(2)
	Si6–Si7: 2.569(6)	Si6–Si7: 2.534(2)	Si3–Si4′: 2.565(2)
	Si5–Si8: 2.585(6)	Si5–Si8: 2.534(2)	Si3′–Si4: 2.565(2)

**Fig. 4:**
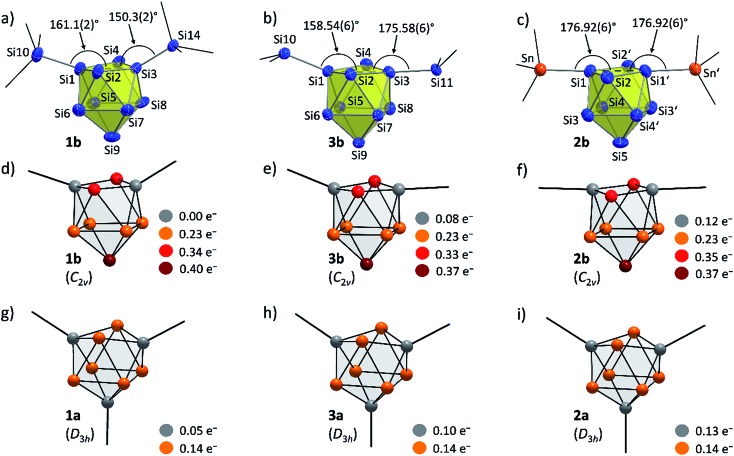
Molecular structures: (a) {Si(TMS)_3_}_2_Si_9_^2–^ (**1b**); (b) (SiH^*t*^Bu_2_)_2_Si_9_^2–^ (**3b**);^30^ (c) (SnCy_3_)_2_Si_9_^2–^ (**2b**, symmetry operation: (′) = –*x*, *y*, 0.5 – *z*); partial atomic charge distributions (DFT-PBE0/TZVP level of theory, all values in e^–^): (d) {Si(TMS)_3_}_2_Si_9_^2–^ (**1b**); (e) (SiH^*t*^Bu_2_)_2_Si_9_^2–^ (**3b**); (f) (SnCy_3_)_2_Si_9_^2–^ (**2b**); (g) {Si(TMS)_3_}_3_Si_9_^–^ (**1a**); (h) (SiH^*t*^Bu_2_)_3_Si_9_^–^ (**3a**); (i) (SnCy_3_)_3_Si_9_^–^ (**2a**). Molecular structures: Si atoms in blue and Sn atoms in red-brown are shown as ellipsoids at 50% probability level; organic groups are shown as black sticks; H_Si_ atoms in **3b** are omitted.

A comparison of the molecular structures of the siliconoid di-anions **1b**, **2b** and **3b** (Fig. 3 and 4) shows specific differences for the arrangement of the two substituents at the respective Si_9_ cluster core. As expected, the longest Si_9_ cluster exo-bonds are detected for the stannyl derivative **3b** with Si_9_–Sn bond lengths of 2.578(1) Å, while the Si_9_–Si cluster exo-bonds in **1b** and **2b** are significantly shorter with values of 2.357(5)/2.339(5) Å (**1b**) and 2.349(2)/2.379(2) Å (**3b**) (Table 1). Si–Sn bonds in low-valent Si-compounds are scarce, but the Si_9_–Sn bond lengths in **2b** compare well to the reported Si–Sn distance in the disilene (Tip)_3_(SnMe_3_)Si_2_ (2.5675(6) Å).^65^ The anion **2b** is located on a twofold symmetry axis, and the two equivalent Sn–Si1–Si1′ and Si1–Si1′–Sn′ angles are 176.92(6)°. In **2b**, the Sn atoms of the SnCy_3_ ligands are almost in plane with the non-capped cluster square formed by the atoms Si1/Si1′/Si2/Si2. In the silyl derivatives **1b** and **3b**, the corresponding angles are smaller, and the exo-bonds are oriented towards the open face of the clusters [**1b**: Si10–Si1–Si3 with 161.1(2)°, Si1–Si3–Si14 with 150.3(2)°; **3b**: Si10–Si1–Si3 with 158.54(6)°, Si1–Si3–Si11 with 175.58(6)°]. The arrangement of the stannyl substituents in **2b** is consistent with the corresponding angles in the di-substituted Ge_9_ derivative (SnPh_3_)_2_Ge_9_^2–^ [corresponding angles: 171.49(5)° and 172.22(5)°] in which one exo-bond is orientated towards the capped face of the cluster.^66^

This indicates a certain degree of interaction of the tin atoms with next-nearest neighbor silicon cluster atoms in **2b** as it was also indicated in the NMR experiments for the tin substituents in the disilene (Tip)_3_(Sn^*t*^Bu_2_Cl)Si_2_.^65^ Such an interaction is further supported by the formation of (SnPh_3_)Ge_9_^3–^ in which the stannyl ligand is bonded to two Ge_9_ cluster atoms.^66^

The highly dispersed ^29^Si NMR signals in solution in a range from –8.70 to –360.72 ppm for **1a** and from –100.01 to –335.50 ppm for **2a** hint for an inhomogeneous electron distribution due to the different oxidation numbers. Such a broad range was observed before in (Tip)_6_Si_6_ (Tip = 2,4,6-triisopropylphenyl) with a tricyclic structure featuring silicon atoms with two, one, and no substituents outside the ring framework. Consequently, (Tip)_6_Si_6_ can be regarded as a tricyclic aromatic isomer of hexasilabenzene.^15^ In contrast to (Tip)_6_Si_6_ with two Si atoms not attached to Tip substituents, bare Si_9_^4–^ clusters reveal nine such atoms. Si_9_^4–^ possesses a fully delocalized electronic system which fits the superatom model of a 40-electron cluster.^67^–^70^ Ligand attachment to Si_9_^4–^ allows for a step-wise transition to molecules with partially delocalized bonds.

In order to investigate the bond properties of the di- and tri-substituted Si_9_ atom clusters we calculated the partial atomic charges [e^–^] of the silicon cluster atoms of **1a**/**1b**, **2a**/**2b** and **3a**/**3b** (Fig. 4).^30^ Comparison of the overall charge distributions shows that lower partial charges are located at substituted silicon atoms including substituent-specific charge differences underlining the electronic influence of the respective substituent on the cluster atoms (partial charges: SnCy_3_ > SiH^*t*^Bu_2_ > Si(TMS)_3_). Interestingly, the partial charges at the unsubstituted Si atoms (prism faces) in the tri-substituted species **1a**/**2a**/**3a** are all identical with a value of 0.14e^–^ and show a homogeneous distribution of the extra negative charge at the cluster prism faces of the *D*_3h_ symmetric cluster. By contrast, the two extra negative charges in the di-substituted *C*_2v_ symmetric clusters **1b**, **2b** and **3b** are distributed more versatilely on the cluster surfaces consisting of ligand-free Si atoms. The partial atomic charges of the silicon atoms in the capped cluster squares [**1b**/**3b**: Si5 to Si8; **2b**: Si3(′) and Si4(′)] are equal for all species with a value of 0.23e^–^ for each atom, whereas the highest partial charges at the square-capping silicon atoms, from which the substituents are detached during crystallization, is found in **1b** (0.40e^–^, Si9 atom).

**Fig. 5:**
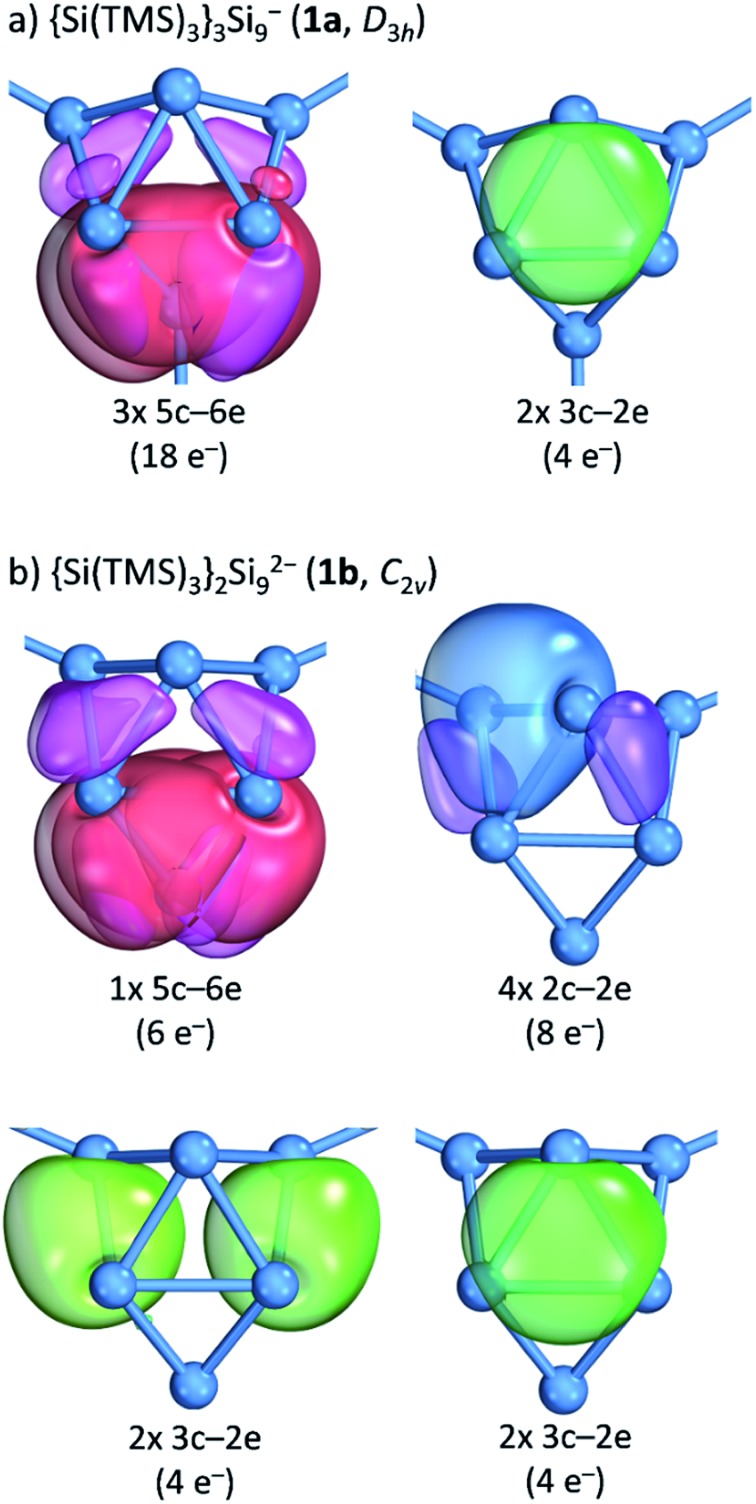
Intrinsic bond orbital (IBO) analysis: (a) the *D*_3h_ symmetric cluster in {Si(TMS)_3_}_2_Si_9_^2–^ (**1a**); (b) the *C*_2v_ symmetric cluster in {Si(TMS)_3_}_2_Si_9_^2–^ (**1b**). The plotted IBO isosurfaces enclose 80% of the total electron density of the IBO (DFT-PBE0/def2-TZVP level of theory). Details to cluster atom distributions to the respective bonds as well as to the 5c-6e bond in **1b** are shown in the ESI.‡

Further insight into the bonding situation within the cluster units is provided by an IBO analysis of **1a** and **1b** (Fig. 5) that manifests an influence of the third substituent on the bonding situation within the Si_9_ cluster cores. The analysis shows a delocalization of the cluster valence electrons (total: 40e^–^) that is in accordance with a previous report for related tris-substituted nona-germanium clusters^71^ stronger for *D*_3h_ symmetric **1a** than for *C*_2v_ symmetric **1b**. Delocalization in **1a** occurs by three 5c-6e (5 center-6 electron) bonds (18e^–^) within the three capped square faces and by two 3c-2e bonds (4e^–^) in the two prism faces. A comparison to the bonding situation in **1b** shows that only one delocalized 5c-6e bond (6e^–^) is present here which is located within the capped square of the cluster from which cap the substituent is released. Most interestingly, causes the attachment of two ligands a high degree of bond localization in form of four covalent 2c-2e bonds (8e^–^) in **1b** in the non-capped square of the cluster. Furthermore, two 3c-2e bonds (total 8e^–^) are present in the triangular faces of **1b**. The remaining 18 cluster valence electrons of **1a** and **1b** are located in the covalent cluster exo-bonds (**1a**: 6e^–^; **1b**: 4e^–^) and in six lone pairs which are distributed over the naked Si cluster atoms (**1a**: 12e^–^, six lone pairs: **1b**: 14e^–^, seven lone pairs). Similar bond delocalization was also reported for hexasilabenzene^15^ in which theoretical analysis revealed the cyclic delocalization of six mobile electrons of the p-, s- and non-bonding type across the central four-membered ring and which was described as dismutational aromatic. The herein presented study to the charge distributions within substituted Si_9_ clusters adds these cluster species as delocalized species to the known silicon molecules in the literature.

**Fig. 6:**
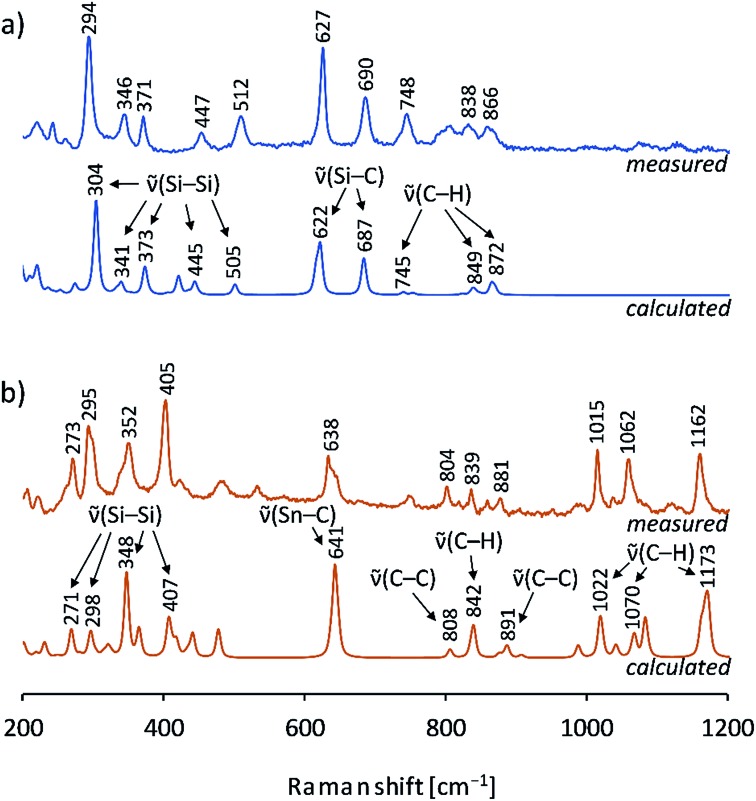
Raman spectra of single crystals: (a) measured spectrum of a (K–222crypt)_2_**1b** single crystal (blue, top), calculated spectrum of {Si(TMS)_3_}_2_Si_9_^2–^ (**1b**) by DFT-PBE0/TZVP (blue, bottom); (b) measured spectrum of a (K–222crypt)_2_**2b** single crystal (red-brown, top), calculated spectrum of (SnCy_3_)_2_Si_9_^2–^ (**2b**) by DFT-PBE0/TZVP (red-brown, bottom); details of the calculations can be found in the ESI.‡

Furthermore, the crystals were characterized by Raman spectroscopy, and the measured vibrations are assigned by comparison to calculated Raman spectra of **1b** and **2b** (Fig. 6). Changes of intensities might be due to specific packing effects (calculations were performed on discrete anionic species) and to orientation effects of the single crystal. In both cases characteristic ν̃(Si–Si) stretching vibrations of the Si_9_ cluster cores were detected which agree well with the calculated values. Interestingly, the peaks and the distribution range of the cluster vibrations are different for the two siliconoids (**1b**: 294, 346, 371, 447 cm^–1^; **2b**: 273, 295, 352, 405 cm^–1^), which indicates an influence of the respective substituent on the Si_9_ intra-bonds. All vibrations for the stannyl derivative **2b** (range: 273–405 cm^–1^) are found at lower wavenumbers than those of **1b** (range: 294–512 cm^–1^). For **1b**, an Si_9_–Si(TMS)_3_ vibration for the cluster exo-bond was found at 512 cm^–1^, whereas the Si_9_–SnCy_3_ exo-bonds are not detectable due to laser absorption effects in the spectrum below 200 cm^–1^. Moreover, ν̃[Si–C] and ν̃[C–H] vibrations were observed for **1b** and ν̃[Sn–C], ν̃[C–C] and ν̃[C–H] vibrations for **2b**, which also include the vibrations for the (K–222crypt) units in the single crystals.

Up to this study, the Raman spectra of Si_9_ cluster compounds comprised the protonated cluster species H_2_Si_9_^2–^ and Si_9_^4–^ that show less Raman vibrations. The spectrum of H_2_Si_9_^2–^ displays a prominent resonance at 386 cm^–1^ and a resonance of Si_9_^4–^ as part of the solid phase K_12_Si_17_ at 390 cm^–1^.^55^ In contrast, multiple Si_9_ vibrations are confirmed for the ligand-stabilized clusters **1b**/**2b**, and a possible correlation with the delocalized bonding situation from the IBO analysis is of interest for future reports.

## Conclusions

The synthetic approach for silicon-rich molecules comprising silicon atoms with low oxidation state from the binary intermetallic precursor K_12_Si_17_ is established. For the first time a metal atom is attached to a Si_9_ cluster core pathway allowing for the structural characterization of Cy_3_Sn–(Si_9_^2–^)–SnCy_3_ (**2b**). In addition the anionic siliconoid {Si(TMS)_3_}_2_Si_9_^2–^ (**1b**) is structurally characterized. Both anions form *via* the tris-substituted derivatives (SnCy_3_)_3_Si_9_^–^ (**1a**) and {Si(TMS)_3_}_3_Si_9_^–^ (**2a**) that have been spectroscopically characterized. In contrast to known “molecular multi-step” approaches, the syntheses became feasible *via* a two-step reaction from elemental silicon. The molecular anions possess high numbers of unsubstituted Si cluster atoms and add as novel delocalized representatives to known silicon compounds in the literature. Moreover, the influence of the ligands on bond localization at the Si_9_ cluster cores was pointed out.

## Experimental section

### General

All reactions and manipulations were performed under a purified argon atmosphere using standard Schlenk and glove box techniques. Thf was dried using the solvent purificator MBraun MB-SPS, and fluorobenzene was dried over CaH_2_ prior to use. 222crypt was dried *in vacuo* prior to use. Liquid ammonia was dried and stored over sodium metal, and all other solvents (including deuterated solvents) were stored over molecular sieves (3 Å). All other chemicals were received commercially and used without further purification.

### K_12_Si_17_ (*activated*)

The *Zintl* compound K_12_Si_17_ was synthesized by heating (heating rate: 2 °C min^–1^) a stoichiometric mixture of 0.55 g K (14.0 mmol, 1 eq.) and 0.59 g Si (21.1 mmol, 1.5 eq.) in a sealed tantalum ampoule to 800 °C for 15 h and subsequent cooling (cooling rate: 0.5 °C min^–1^) to room temperature. The ampoule was opened in a glove box, and the product was finely ground yielding 1.04 g K_12_Si_17_ (1.10 mmol, 94%) as a black solid. The solid was characterized by powder X-ray diffraction showing high purity for the solid phase (ESI‡). For the activation, 0.20 g K_12_Si_17_ (0.21 mmol, 1 eq.) and 0.15 g 222crypt (0.39 mmol, 1.9 eq.; 4,7,13,16,21,24-hexaoxa-1,10-diazabicyclo[8.8.8]hexacosan) were weighed into a Schlenk tube, and liquid ammonia was added at –78 °C (iPrOH/CO_2_) to give a dark red solution. The solution was stirred at –78 °C for 2 h, and K_12_Si_17_ (*activated*) was obtained as a brown solid after the removal of liquid ammonia.

### (K–222crypt)^+^ salts of **1a** and **1b**

K_12_Si_17_*activated* (batch with 200 mg K_12_Si_17_) was cooled to 0 °C (ice bath), and thf (12 mL) was added. 0.36 g Si(TMS)_3_Cl (1.26 mmol, 6 eq.) was added under continuous stirring as a pre-cooled thf solution (3 mL). The reaction mixture was stirred overnight and allowed to warm to room temperature. A brownish filtrate was obtained after filtration, and all volatiles were removed *in vacuo*. The residue was extracted with fluorobenzene (5 mL), and a red filtrate was obtained. After washing with hexane and vacuum drying, a light brown solid containing the (K–222crypt)^+^ salt of **1a** was obtained (yield: 0.12 g, 40% based on K_12_Si_17_). The solid was characterized by ESI-MS in thf and NMR spectroscopy (^1^H, ^13^C, ^29^Si) in solution (thf-*d*_8_). For crystallization, a fluorobenzene solution of the solid (3 mL) was layered with hexane. Yellow block-shaped crystals of the (K–222crypt)^+^ salt of **1b** suitable for single crystal X-ray diffraction were obtained after 10 d (10 mg, 10% based on (K–222crypt)**1a**). The crystals were investigated by Raman spectroscopy, and the K : Si ratio was confirmed by EDX analyses on single crystals.

#### Bulk material containing **1a**

ESI-MS (negative mode, 4500 V, 300 °C): *m*/*z* = 996 {Si(TMS)_3_}_3_Si_9_^–^; ^1^H NMR (500 MHz, thf-*d*_8_) *δ* 3.64 (s, 12H, O–C*H*_2_–C*H*_2_–O_222crypt_), 3.59 (t, *J* = 5.05 Hz, 12H, O–C*H*_2_–CH_2_–N_222crypt_), 2.61 (t, 12H, *J* = 5.10 Hz, O–CH_2_–C*H*_2_–N_222crypt_), 0.25 (s, 81H, Si*Me*_3_); ^13^C{^1^H} NMR (75 MHz, thf-*d*_8_) *δ* 71.47 (O–*C*H_2_–*C*H_2_–O_222crypt_), 67.44 (O–*C*H_2_–CH_2_–N_222crypt_), 55.01 (O–CH_2_–*C*H_2_–N_222crypt_), 3.51 (Si*Me*_3_); ^29^Si{^1^H} NMR (99 MHz, thf-*d*_8_) *δ* –8.70 [Si(*TMS*)_3_], –129.94 [*Si*(TMS)_3_], –175.29 [*Si*–Si(TMS)_3_], –360.72 [*Si*–Si–Si(TMS)_3_].

#### Single crystals containing **1b**

EDX analysis [single crystals of (K–222crypt)_2_**1b**]: K : Si = 6.30% : 93.7% (calcd: 6.22% : 93.8%); Raman (532 nm): 294 [ν̃(Si–Si) calcd 304], 346 [ν̃(Si–Si) calcd 341], 371 [ν̃(Si–Si) calcd 373], 447 [ν̃(Si–Si) calcd 445], 512 [ν̃(Si–Si) calcd 505], 627 [ν̃(Si–C) calcd 622], 690 [ν̃(Si–C) calcd 687], 748 [ν̃(C–H) calcd 745], 838 [ν̃(C–H) 849], 866 [ν̃(C–H) calcd 872] cm^–1^.

### (K–222crypt)^+^ salts of **2a** and **2b**

K_12_Si_17_*activated* (batch with 200 mg K_12_Si_17_) was cooled to 0 °C (ice bath), and pyridine (12 mL) was added. 0.51 g (1.26 mol, 6 eq.) SnCy_3_Cl was added under continuous stirring as pre-cooled pyridine solution (3 mL). The reaction mixture was stirred overnight and filtered under continuous cooling yielding a colored filtrate which was dried *in vacuo* and extracted with fluorobenzene (5 mL). The red filtrate was dried *in vacuo*, and a brown solid containing the (K–222crypt)^+^ salt of **2a** was obtained (yield: 0.10 g, 27% based on K_12_Si_17_). The solid was investigated by ESI-MS in thf and NMR spectroscopy (^1^H, ^13^C, ^29^Si, ^119^Sn) in solution (thf-*d*_8_). For crystallization, a fluorobenzene solution of the solid (3 mL) was layered with hexane. Yellow block-shaped crystals of the (K–222crypt)^+^ salt of **2b** suitable for single crystal X-ray diffraction were obtained after 12 d (7 mg, 7% based on (K–222crypt)**2a**). The crystals were investigated by Raman spectroscopy, and the K : Si ratio was confirmed by EDX analyses on single crystals.

#### Bulk material containing **2a**

ESI-MS (negative mode, 4500 V, 300 °C): *m*/*z* = 1357 (SnCy_3_)_3_Si_9_^–^, *m*/*z* = 996 (SnCy_3_)_2_Si_9_^–^; ^1^H NMR (400 MHz, thf-*d*_8_) *δ* 3.67 (s, 12H, O–C*H*_2_–C*H*_2_–O_222crypt_), 3.61 (s, 12H, O–C*H*_2_–CH_2_–N_222crypt_), 2.61 (s, 12H, O–CH_2_–C*H*_2_–N_222crypt_), 1.94–1.30 (m, 69H, *H*_cyclohexyl_); ^13^C{^1^H} NMR (126 MHz, thf-*d*_8_) *δ* 71.58 (O–*C*H_2_–*C*H_2_–O_222crypt_), 68.71 (O–*C*H_2_–CH_2_–N_222crypt_), 55.09 (O–CH_2_–*C*H_2_–N_222crypt_), 33.96 (*C*_cyclohexyl_), 31.10 (*C*_cyclohexyl_), 25.86 (*C*_cyclohexyl_), 21.54 (*C*_cyclohexyl_); ^29^Si{^1^H} NMR (99 MHz, thf-*d*_8_) *δ* –100.01 [*Si*–Sn(Cy)_3_], –335.50 [*Si*–Si–Sn(Cy)_3_]; ^119^Sn{^1^H} NMR (112 MHz, thf-*d*_8_) *δ* –70.50 [*Sn*(Cy)_3_].

#### Single crystals containing **2b**

EDX analysis [single crystals of (K–222crypt)_2_**2b**]: K : Si : Sn = 15.5% : 44.1% : 40.4% (calcd: 13.8% : 44.5% : 41.8%); Raman (785 nm):, 273 [ν̃(Si–Si) calcd 271], 295 [ν̃(Si–Si) calcd 298], 352 [ν̃(Si–Si) calcd 348], 405 [ν̃(Si–Si) calcd 407], 638 [ν̃(Sn–C) calcd 641], 804 [ν̃(C–C) calcd 808], 839 [ν̃(C–H) calcd 842], 881 [ν̃(C–C) calcd 891], 1015 [ν̃(C–H) calcd 1022], 1062 [ν̃(C–H) calcd 1070], 1162 [ν̃(C–H) calcd 1173] cm^–1^.

### Computational details

Quantum-chemical calculations at the DFT-PBE0/TZVP level of theory were carried out using the TURBOMOLE program package.^72^–^76^ Intrinsic atomic orbitals (IAOs) and intrinsic bond orbitals were used to analyze the partial charges and bonding of the clusters, respectively.^77^ Full computational details are available in the ESI.‡

### Single crystal structure determination

For single crystal data collection, the crystals were fixed on a glass capillary and positioned in a cold stream of N_2_ gas. Single crystal data collection was performed with a STOE StadiVari (Mo Kα radiation) diffractometer equipped with a DECTRIS PILATUS 300K detector. Structures were solved by Direct Methods (SHELXS-2014) and refined by full-matrix least-squares calculations against *F*^2^ (SHELXL-2014).^78^ The positions of the hydrogen atoms were calculated and refined using a riding model. Unless otherwise stated, all non-hydrogen atoms were treated with anisotropic displacement parameters. In **1b** some hypersilyl groups show rotational disorder and were refined at two split positions. In **2b**, one cyclohexyl group is found in two different orientations and was refined at two split positions. For visualization, the crystal structures have been plotted with Diamond.‡ 79

### Electron dispersive X-ray (EDX) analysis

Single crystals of all compounds were analyzed with a SWIFT-ED-TM (Oxford Instruments) and a Hitachi TM-1000 Tabletop microscope (Hitachi High-Technologies) with the INCA system software.

### NMR spectroscopy

^1^H, ^13^C and ^29^Si NMR spectra were recorded on a Bruker AVIII Ultrashield 400 MHz or a Bruker AVIII 500 MHz Cryo system, ^119^Sn NMR spectra were measured on a Bruker AVIII 300 MHz (*Bruker Inc*) instrument. The signals of the ^1^H and ^13^C spectra were calibrated on the rest proton signal of the used deuterated solvent thf-*d*_8_. Chemical shift values are given in *δ* values by parts per million (ppm). The coupling constants *J* are stated in Hz. Signal multiplicities are abbreviated as follows: s – singlet, d – doublet, t – triplet, m – triplet. The spectra were evaluated with MestReNova.^80^

### Electrospray ionization mass spectrometry (ESI-MS)

The preparation of the samples was done in a glove box. The spectra were measured on an *HCT* instrument (*Bruker Inc*). The data were analyzed using the program *Bruker Compass Data Analysis 4.0 SP 5*. The dry gas temperature was adjusted to 300 °C and the injection speed to 240 μL s^–1^. For fragmentation experiments, the respective mass peaks were isolated (width: 40) and fragmented (amplitude: 2.0). Visualization of the spectra was carried out with the programs *OriginPro* (*Origin Lab Inc*) or *Microsoft Excel* (*Microsoft Inc*).

### Raman spectroscopy

Raman spectra were recorded with an *inVia* Raman Microscope *RE04* with a *CCD* detector and 500 mW maximal power (Renishaw PLC; Software: *WiRE 4.2 build 5037*) at *λ* = 532 nm. For the measurements the samples were sealed in glass capillaries in a glove box.

### Powder X-ray diffraction (PXRD)

The data were collected at room temperature on a STOE Stadi P diffractometer (Ge(111) monochromator, Cu Kα_1_ radiation, *λ* = 1.54056 Å) with a Dectris MYTHEN 1K detector in Debye–Scherrer geometry. For the measurements the samples were sealed in glass capillaries (*Ø* 0.5 mm). The raw data were processed with *WinX-POW*,^81^*OriginPro* (*Origin Lab Inc*) was used for the visualization.

## Conflicts of interest

There are no conflicts to declare.

## Supplementary Material

Supplementary information

Crystal structure data
