# Supplementary material for: Silicon clusters with six and seven unsubstituted vertices via a two-step reaction from elemental silicon
Source: Chem Sci. 2019 Aug 15;10(39):9130–9. doi: 10.1039/c9sc03324f (PMC6889835; doi:10.1039/c9sc03324f)
Supplement: Supplementary file 1 [file SC-010-C9SC03324F-s001.pdf]

Electronic Supplementary Material (ESI) for Chemical Science.
This journal is © The Royal Society of Chemistry 2019

Silicon Clusters with Six and Seven Unsubstituted Vertices *via* a Two-step Reaction from Elemental Silicon

Supporting Information

1. PXRD of the *Zintl* phase precursor $K_{12}Si_{17}$
2. ESI-MS spectra
3. NMR spectra
4. Crystallographic details
5. Computational details
6. References

1. PXRD of the Zintl phase precursor $K_{12}Si_{17}$

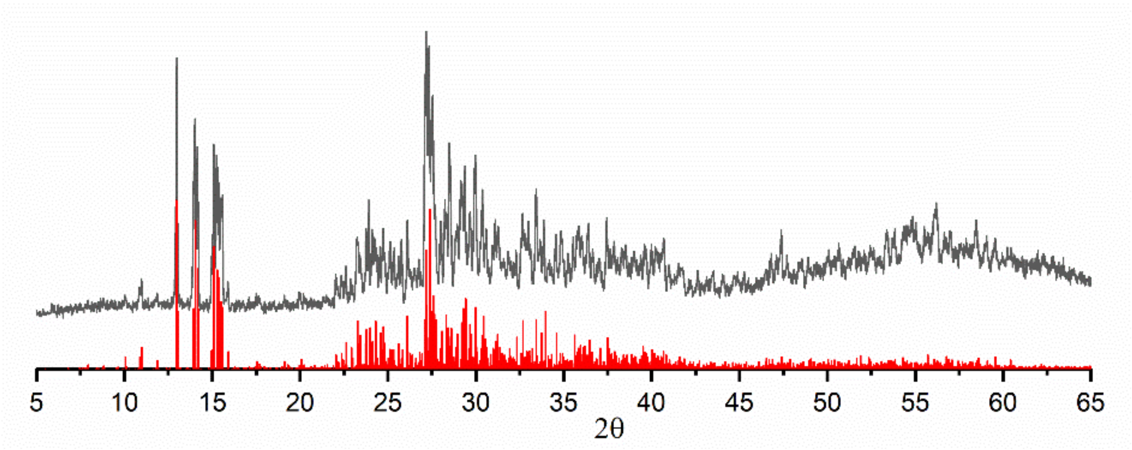

Figure SI 1. Powder X-ray diffractogram of the Zintl phase precursor $K_{12}Si_{17}$ (gray: *measd.*; red: *calcd.* from single crystal data).[1]

2. ESI-MS spectra

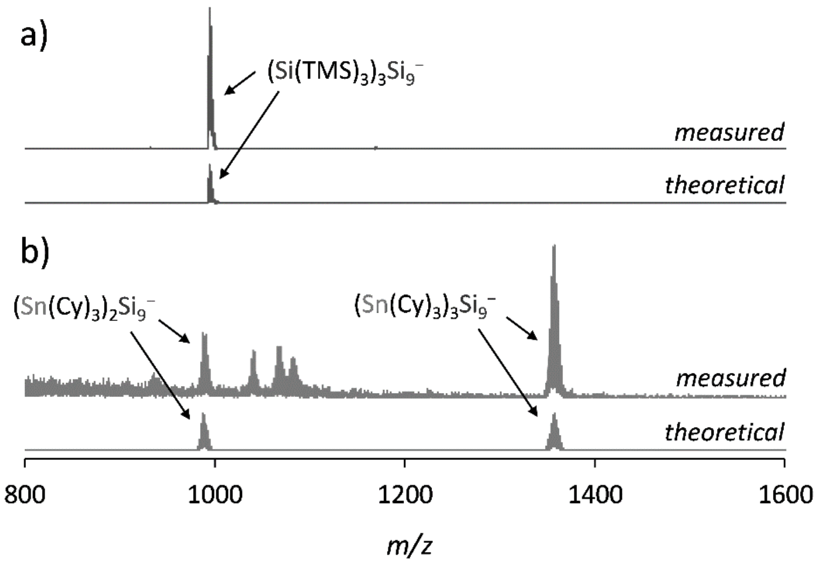

Figure SI 2. a) ESI-MS spectrum of the bulk material containing **1a** in thf (*m/z* = 996, **1a**); b) ESI-MS spectrum of the bulk material containing **2a** in pyridine (*m/z* = 1357, **2a**; *m/z* = 989, **2b**).

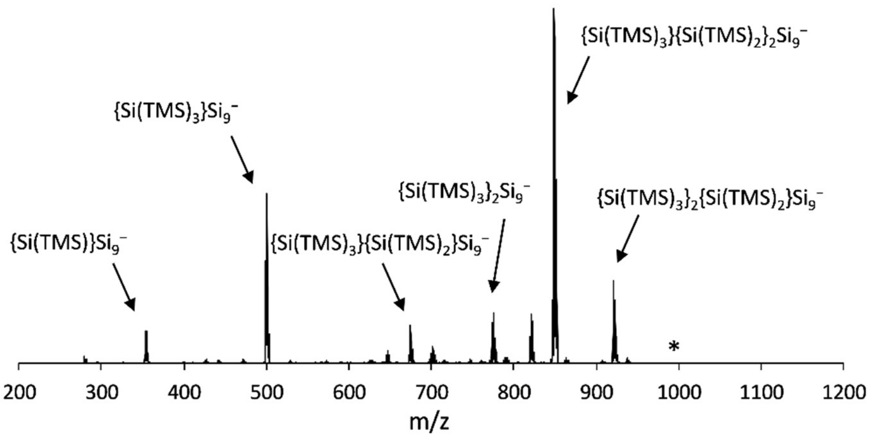

Figure SI 3. ESI-MS fragmentation spectrum of the $\{Si(TMS)_3\}_3Si_9^-$ (**1a**) mass peak (fragmented mass at *m/z* = 996: *) in thf; negative mode, 4000 V, 300 °C.

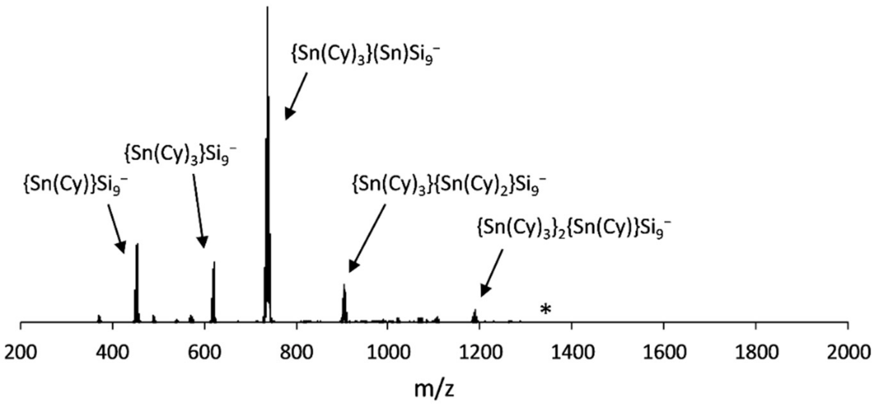

Figure SI 4. ESI-MS fragmentation spectrum of the $(SnCy_3)_3Si_9^-$ (**2a**) mass peak (fragmented mass at *m/z* = 1357: *) in pyridine; negative mode, 4500 V, 300 °C.

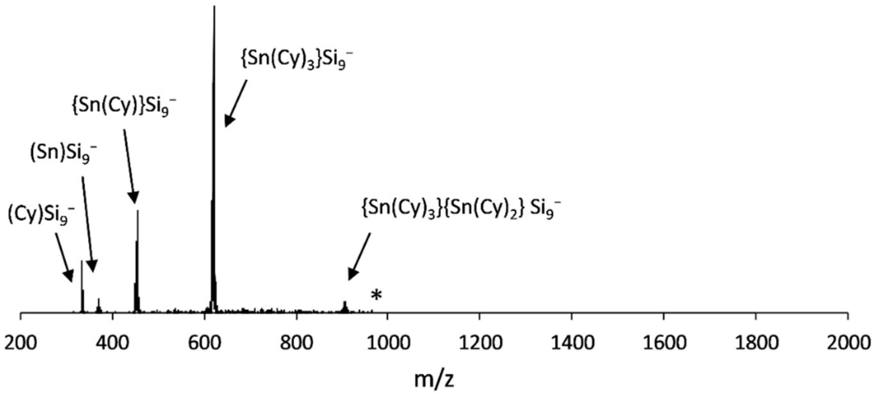

Figure SI 5. ESI-MS fragmentation spectrum of the $(SnCy_3)_2Si_9^-$ mass peak (fragmented mass at *m/z* = 989: *) in pyridine; negative mode, 3500 V, 300 °C.

3. NMR spectra

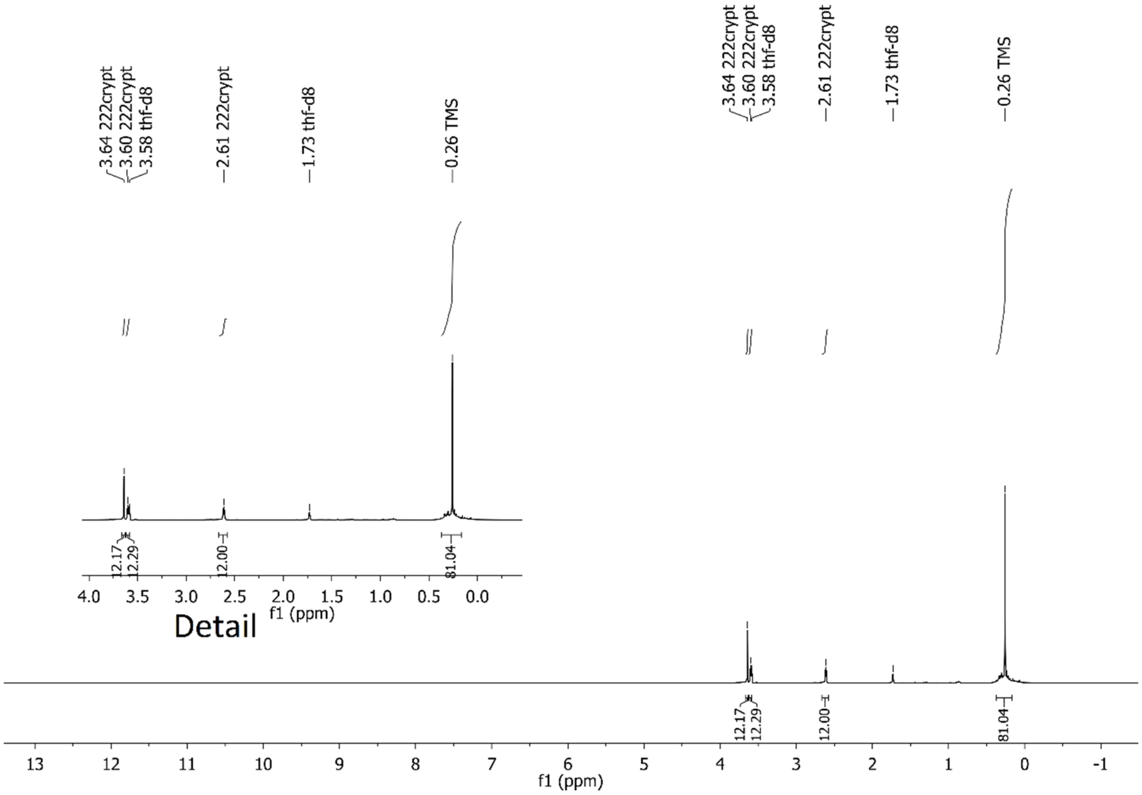

Figure SI 6. ^{1}H NMR (thf-*d8*) spectrum of the bulk material containing **1a**.

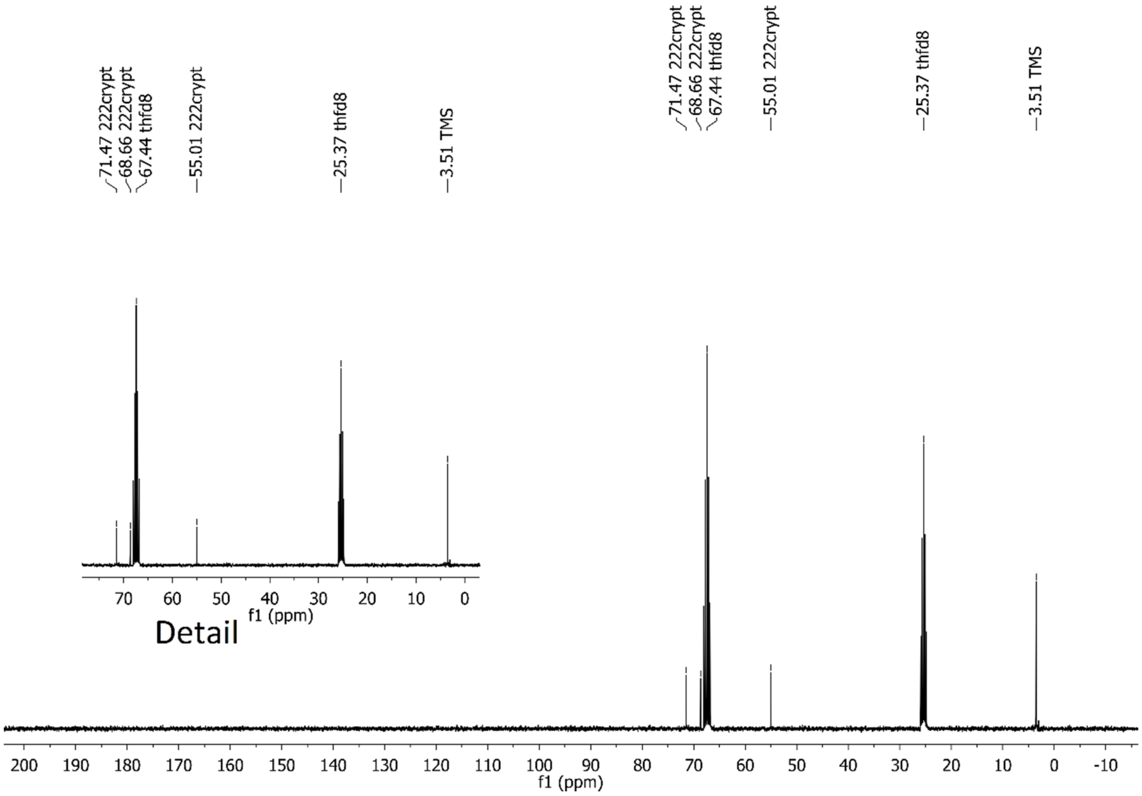

Figure SI 7. $^{13}C\{^{1}H\}$ NMR (thf-*d8*) spectrum of the bulk material containing **1a**.

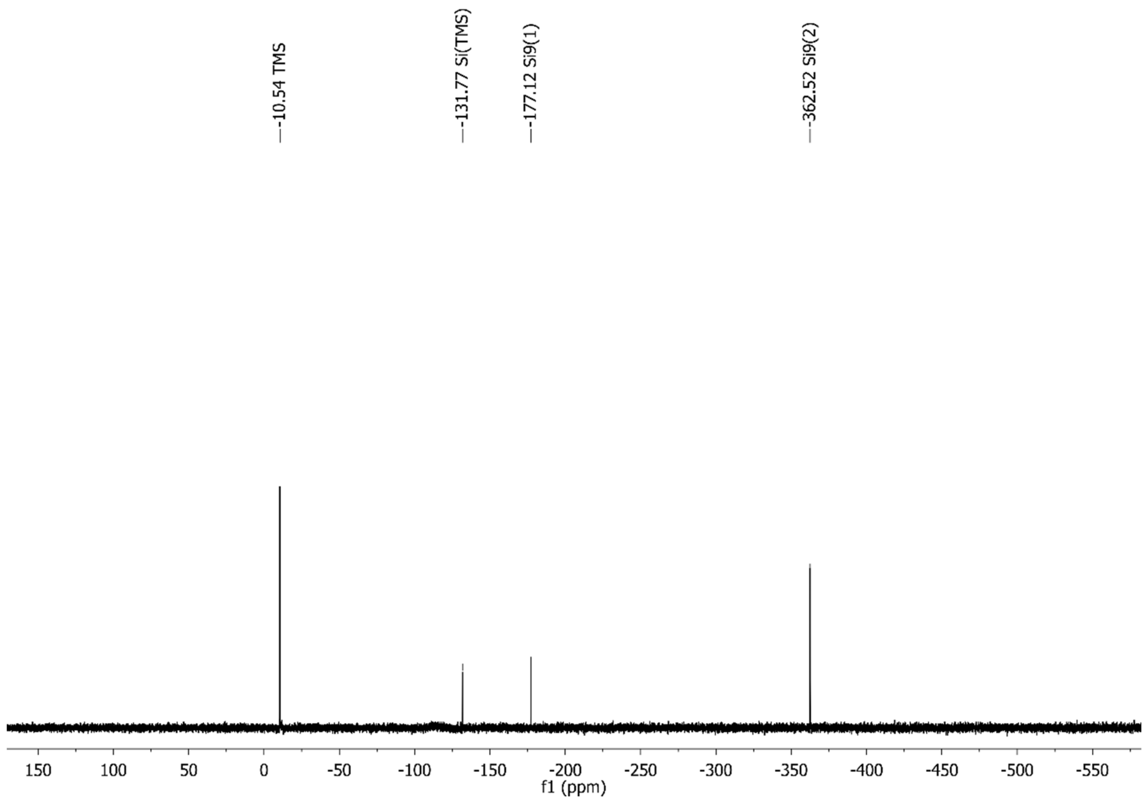

Figure SI 8. $^{29}Si\{^{1}H\}$ NMR (thf-*d8*) spectrum of the bulk material containing **1a**.

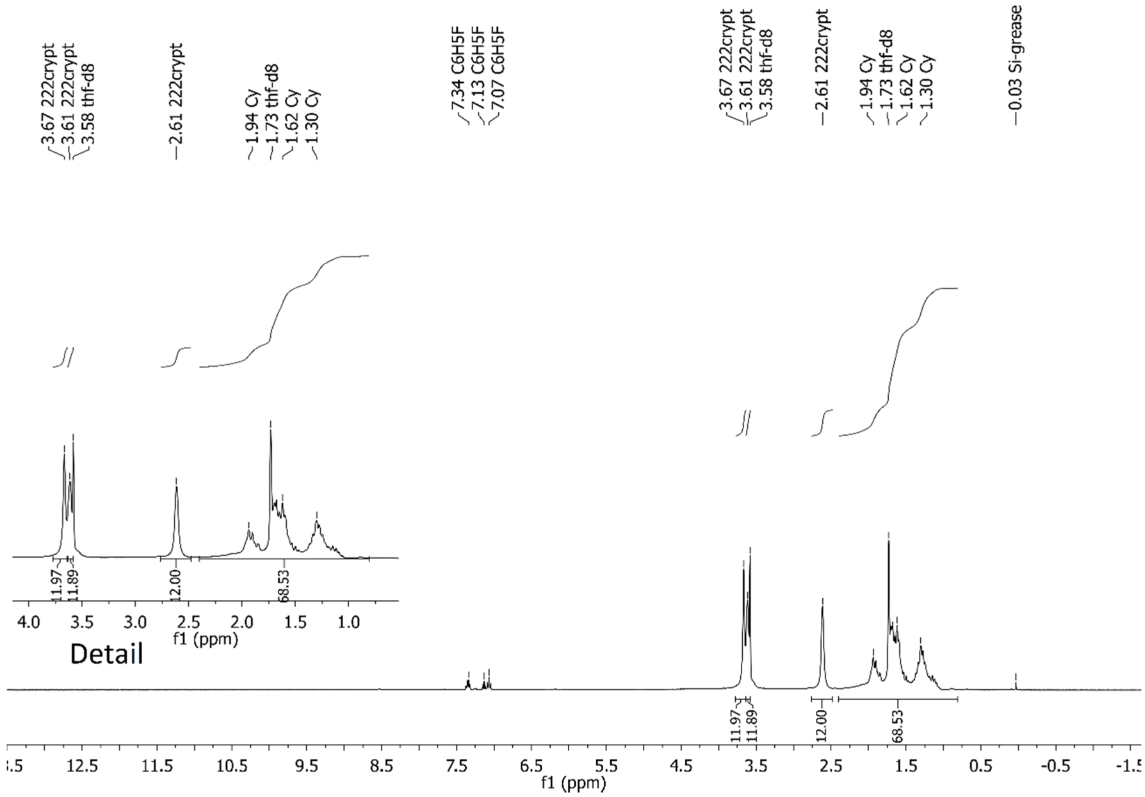

Figure SI 9. ^{1}H NMR (thf-*d8*) spectrum of the bulk material containing **2a**.

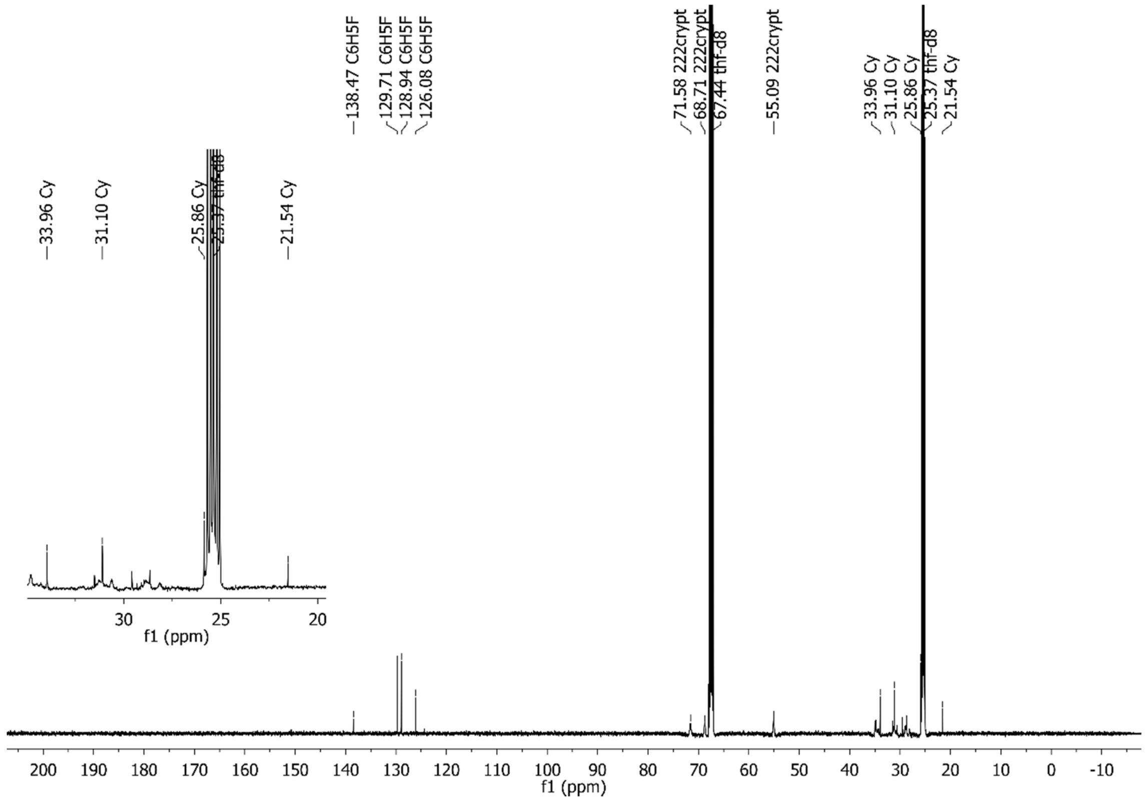

Figure SI 10. $^{13}C\{^{1}H\}$ NMR (thf-*d8*) spectrum of the bulk material containing **2a**.

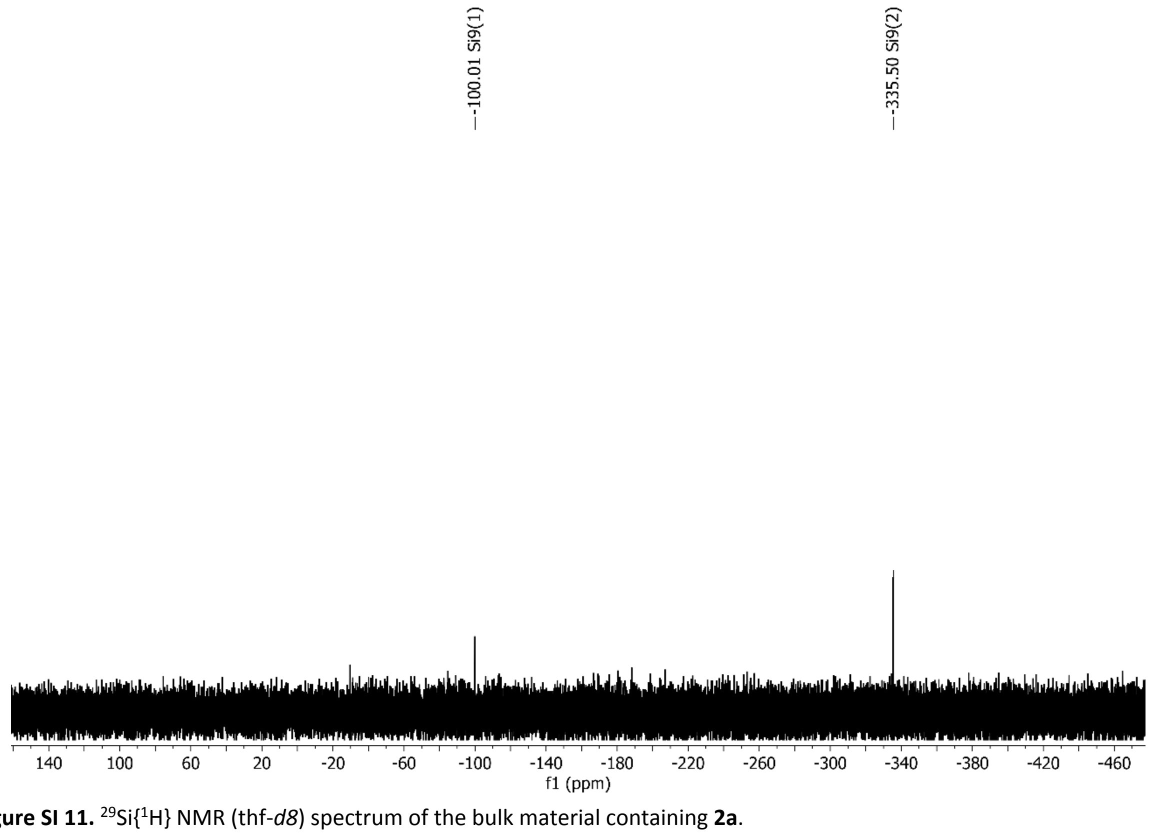

Figure SI 11. $^{29}Si\{^{1}H\}$ NMR (thf-*d8*) spectrum of the bulk material containing **2a**.

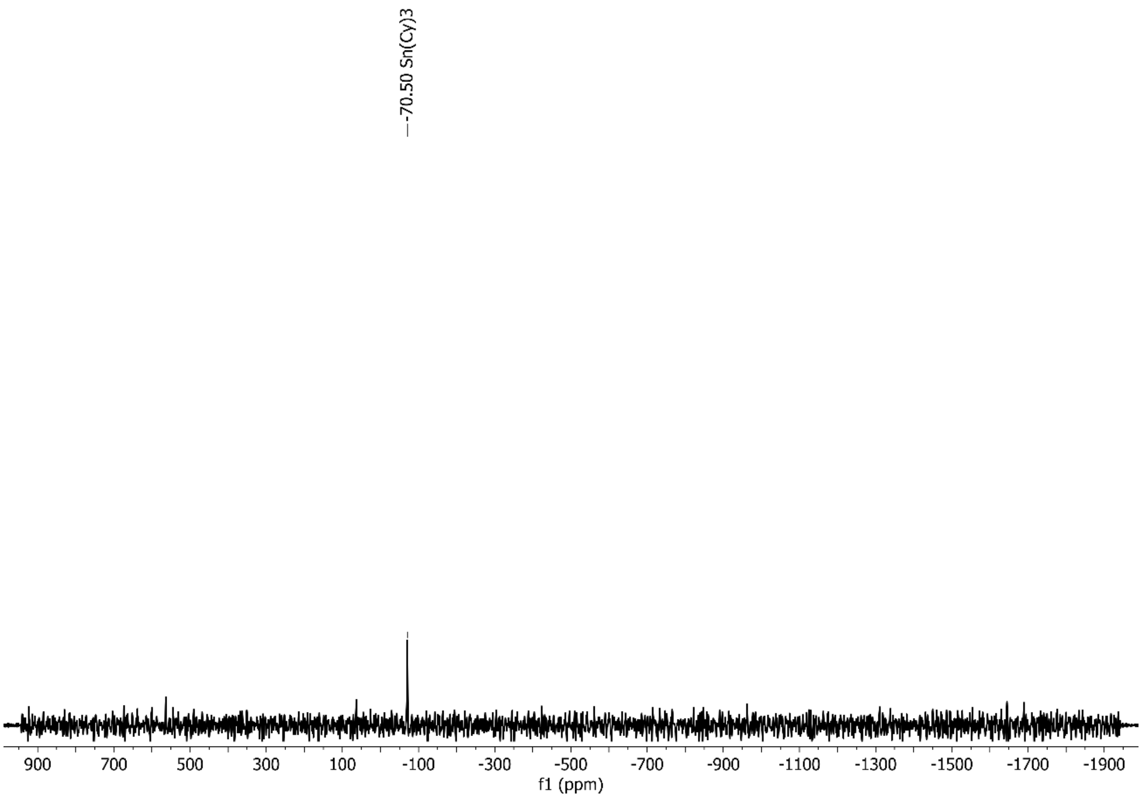

Figure SI 12. $^{119}Sn\{^{1}H\}$ NMR (thf-*d8*) spectrum of the bulk material containing **2a**.

4. Crystallographic details

Table SI 1. Crystallographic data and details of the structure determinations of (K-222crypt)$_2$**1b** and (K-222crypt)$_2$**2b**.

**Compound**	**(K-222crypt)$_2$1b ·C_6H_5F**	**(K-222crypt)$_2$2b**
formula	$C_{60}H_{131}FK_2N_4O_{12}Si_{17}$	$C_{72}H_{138}K_2N_4O_{12}Si_9Sn_2$
crystal shape	yellow block	yellow block
crystal size [mm]	0.4 x 0.2 x 0.3	0.4 x 0.2 x 0.1
fw (g·mol^{-1})	1675.41	1820.25
space group (no)	P_n (7)	*C*2/*c* (15)
a (Å)	16.4939(9)	31.6324(14)
b (Å)	25.3561(17)	11.8423(7)
c (Å)	22.6197(12)	25.7817(12)
α (deg)	90	90
β (deg)	90.756(4)	108.231(3)
γ (deg)	90	90
V (Å^3)	9459.2(10)	9173.0(8)
Z	4	4
T (K)	120(2)	150(2)
ρ_{calc} (g·cm^{-3})	1.176	1.318
μ (mm^{-1})	0.366	0.806
measured reflections	60136	97474
R_{int}	0.0524	0.1068
hkl range	$-20 < h < 20$<br>$-27 < k < 29$<br>$-26 < l < 27$	$-38 < h < 38$<br>$-14 < k < 14$<br>$-31 < l < 31$
2 θ range	5.416 – 52.000	5.188 – 51.994
independent reflections	24142	9014
reflections [$I > 2\,\sigma(I)$]	12835	5313
parameters / restraints	1892 / 422	511 / 0
R_1 [$I > 2\,\sigma(I)$ / all data]	0.0729 / 0.1411	0.0444 / 0.0978
wR_2 [$I > 2\,\sigma(I)$ / all data]	0.1606 / 0.1880	0.0836 / 0.0997
goodness of fit	0.932	0.928
largest difference peak/hole [e Å^{-3}]	0.925 / −0.448	1.074 / −0.365
CCDC number	1896557	1896556

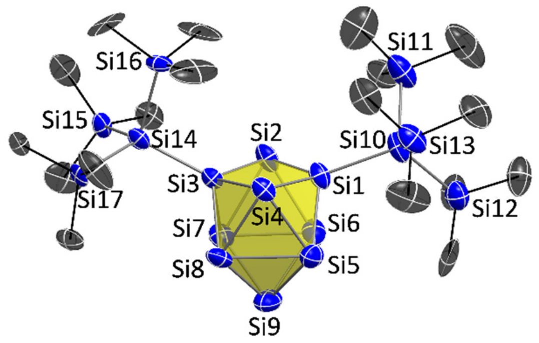

Figure SI 13. Molecular structure of $\{Si(TMS)_3\}_2Si_9^{2-}$ (**1b**) from the single crystal structure determination: Si and C atoms (in blue and black, respectively) are shown as ellipsoids at 50% probability level, H atoms are omitted, minor occupations of the disordered silyl groups are not shown.

Table SI 2. Selected interatomic distances from the single crystal structure determination of $(K\text{-}222crypt)_2$**1b**.

Atom 1	Atom 2	Distance [Å]	Atom 1	Atom 2	Distance [Å]
Si1	Si2	2.396(5)	Si4	Si8	2.488(5)
Si2	Si3	2.395(4)	Si4	Si5	2.485(6)
Si3	Si4	2.398(5)	Si5	Si6	2.772(6)
Si4	Si1	2.427(5)	Si6	Si7	2.569(6)
Si1	Si3	2.992(6)	Si7	Si8	2.738(5)
Si2	Si4	3.759(6)	Si8	Si5	2.585(6)
Si1	Si5	2.415(6)	Si5	Si9	2.468(6)
Si1	Si6	2.425(5)	Si6	Si9	2.470(6)
Si2	Si6	2.446(6)	Si7	Si9	2.427(6)
Si2	Si7	2.521(5)	Si8	Si9	2.475(7)
Si3	Si7	2.418(5)	Si1	Si10	2.357(5)
Si3	Si8	2.401(5)	Si3	Si14	2.339(5)

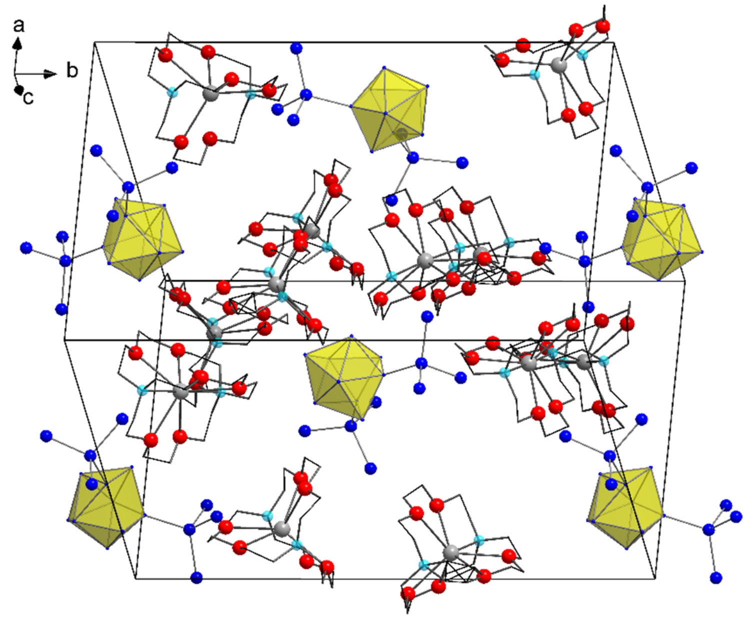

Figure SI 14. Extended unit cell of (K-222crypt)$_2$**1b** [Si_9 clusters are shown as yellow polyhedra, the Si atoms of the $Si(TMS)_3$ substituents (blue), K atoms (gray), O atoms (red), and N atoms (turquoise) are shown as balls, C atoms of 222crypt are shown as wire-sticks, TMS groups and H atoms of 222crypt are omitted].

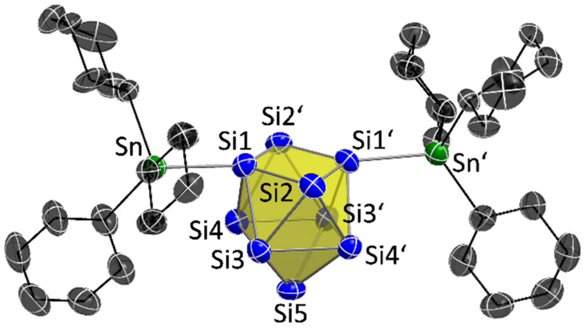

Figure SI 15. Molecular structure of $(SnCy_3)_2Si_9^{2-}$ (**2b**) from the single crystal structure determination: Si, Sn and C atoms (blue, green and black, respectively) are shown as ellipsoids at 50% probability level, H atoms are omitted, minor occupation of the disordered Cy group is not shown, symmetry operation (') −x, y, 0.5−z.

Table SI 3. Selected interatomic distances from the single crystal structure determination of (K-222crypt)$_2$**2b**.

Atom 1	Atom 2	Distance [Å]	Atom 1	Atom 2	Distance [Å]
Si1	Si2	2.430(2)	Si2'	Si4	2.466(2)
Si2	Si1'	2.433(2)	Si3	Si4	2.664(2)
Si1'	Si2'	2.430(2)	Si3'	Si4	2.565(2)
Si2'	Si1	2.433(2)	Si3'	Si4'	2.664(2)
Si1	Si4	2.457(2)	Si3	Si4'	2.565(2)
Si1	Si3	2.456(2)	Si4	Si5	2.436(2)
Si2	Si3	2.478(2)	Si3	Si5	2.432(2)
Si2	Si4'	2.466(2)	Si4'	Si5	2.436(2)
Si1'	Si4'	2.457(2)	Si3'	Si5	2.432(2)
Si1'	Si3'	2.456(2)	Si1	Sn	2.578(1)
Si2'	Si3'	2.478(2)	Si1'	Sn'	2.578(1)

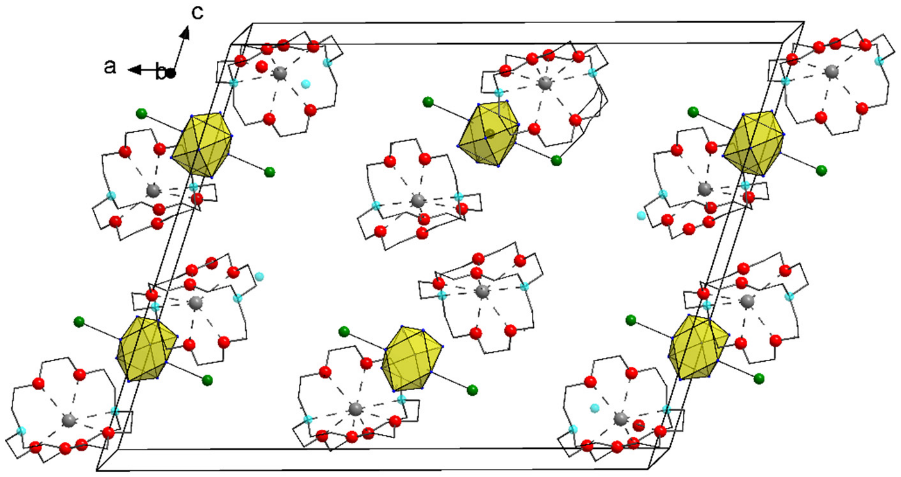

Figure SI 16. Extended unit cell of (K-222crypt)$_2$**2b** [Si_9 clusters are shown as yellow polyhedra, Sn atoms (green), K atoms (gray), O atoms (red), and N atoms (turquoise) are shown as balls, C atoms of 222crypt are shown as wire-sticks, Cy groups and H atoms of 222crypt are omitted].

5. Computational details

Quantum chemical calculations were carried out using the TURBOMOLE program package.[2, 3] We used the PBE0 hybrid density functional method[4, 5] and a triple-zeta-valence quality basis set with polarization functions (def2-TZVP for Si and Sn, def-TZVP for C and H).[6] Multipole-accelerated resolution-of-the-identity technique was used to speed up the calculations.[7-9] The COSMO continuum solvation model was used to counter the twofold negative charge of the dianions.[10] The molecular structures of the di- and tri-substituted clusters were fully optimized within C_{2v} and C_{3v} point group symmetry, respectively. In the 0 K calculations, the symmetry of the tri-substituted clusters is reduced from the ideal D_{3h} to C_{3v} because the ligands cannot rotate freely, but this does not affect the interpretation of the results. A hypothetical tetra-substituted cluster $\{Si(TMS)_3\}_4Si_9$ was optimized without any symmetry constraints. The optimized structures of the studied systems in XYZ format are reported below. Harmonic frequency calculations were carried out to confirm that the structures are true local minima (numerical frequency calculation with the COSMO solvent model). The structure **1b** had three imaginary vibrational modes corresponding to a rotation of methyl and TMS groups (140*i*, 63*i*, 55*i*). The rotational imaginary modes disappear by lowering the point group symmetry to C_2 (the ligands retain the same overall orientation as in the C_{2v} point group). In the Raman spectrum calculations (T = 298.15 K, experimental laser wavelength, unpolarized radiation, scattering angle of 90°),[11, 12] the COSMO solvent model was not used in the dynamic polarizability derivatives calculation. The Raman intensities are given relative to the most intensive peak. The harmonic frequencies of **2b** were scaled by a factor of 0.985 to facilitate comparisons with the experimental spectrum. The final Raman spectra were convoluted using Lorenzian peak profiles with FWHM of 10 cm^{-1}. The peak assignment was carried out by visual inspection of the normal modes (Jmol program package[13]). Intrinsic Atomic Orbitals (IAOs) and Intrinsic Bond Orbitals (IBO) were used to analyze the partial charges and bonding of the clusters, respectively.[14]

Table SI 4. Analysis of the Intrinsic Bond Orbitals (IBO) for **1a**. For each IBO, the atoms forming the IBO and their contributions in percentages are shown (in most cases, the contributions do not add up to 100%, because small contributions from other atoms are not listed separately). Each IBO contains two electrons. For the numbering scheme, see the figure below ([a] not a cluster atom).

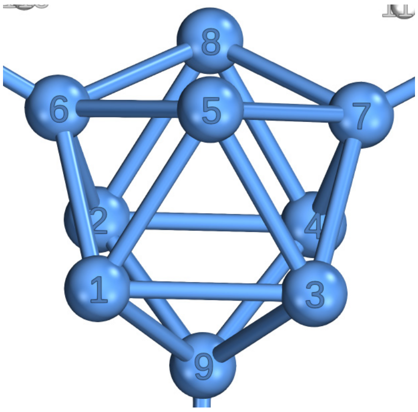

**Atom 1**	**%**	**Atom 2**	**%**	**Atom 3**	**%**
Lone pairs (12 electrons in total)					
1	91				
2	91				
4	91				
5	91				
8	91				
9	91				
Exo-bonds (6 electrons in total)					
9	51	[a]	46		
7	51	[a]	46		
6	51	[a]	46		
3c–2e bonds in trigonal prism faces (4 electrons in total)					
5	33	3	33	1	33
8	33	2	33	4	33
5c–6e system of the three trigonal prism faces and their capping atoms (18 electrons in total)					
6	46	2	31	8	18
6	49	5	33	8	9
6	47	1	35	8	9

Table SI 5. Analysis of the Intrinsic Bond Orbitals (IBO) for **1b**. For each IBO, the atoms forming the IBO and their contributions in percentages are shown (in most cases, the contributions do not add up to 100%, because small contributions from other atoms are not listed separately). Each IBO contains two electrons. For the numbering scheme, see the figure below ([a] not a cluster atom).

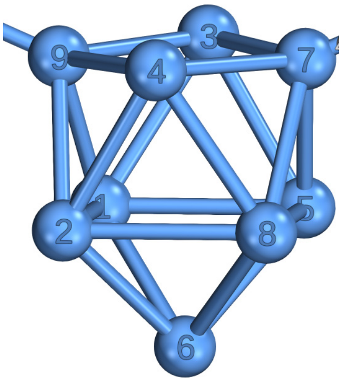

**Atom 1**	**%**	**Atom 2**	**%**	**Atom 3**	**%**
Lone pairs (14 electrons in total)					
1	94				
2	93				
3	93				
4	92				
5	92				
6	92				
8	92				
Exo-bonds (4 electrons in total)					
9	51	[a]	46		
7	51	[a]	46		
3c–2e bonds with one open-square atom and two closed square atoms (8 electrons in total)					
7	41	5	29	8	28
9	42	1	29	2	27
3	34	5	32	1	32
4	36	8	31	2	29
2c–2e bonds in open square, minor contribution from one closed square atom (8 electrons in total)					
9	48	4	39	2	6
7	48	4	39	8	6
7	49	3	39	5	5
9	49	3	39	1	5
5c–6e system of cap atom and the closed square (6 electrons in total)					
6	38	8	36	2	19
6	39	1	36	2	16
6	38	5	38	2	8

Table SI 6. Optimized XYZ coordinates of the studied systems [Å].

$\{Si(TMS)_3\}_2Si_9^{2-}$ (**1b**)				$(SnCy_3)_2Si_9^{2-}$ (**2b**)				$\{Si(TMS)_3\}_4Si_9$			
Si	-1.2743517	1.3358186	-3.1399453	Si	-1.2753634	1.344217	-2.7316151	Si	-0.3138832	0.7008659	1.0506022
Si	-1.2743517	-1.3358186	-3.1399453	Si	-1.2753634	-1.344217	-2.7316151	Si	-0.5741312	0.7450059	-2.5078788
Si	0	1.8459512	-1.0760077	Si	0	1.8552706	-0.6760474	Si	-0.5750799	-1.7152433	0.7111365
Si	0	-1.8459512	-1.0760077	Si	0	-1.8552706	-0.6760474	Si	-0.756453	-1.6634308	-2.0380319
Si	1.2743517	1.3358186	-3.1399453	Si	1.2753634	1.344217	-2.7316151	Si	1.7971899	-0.4427737	0.5577963
Si	0	0	-4.7236547	Si	0	0	-4.3102175	Si	1.0184658	1.4641647	-0.812898
Si	1.5434157	0	-1.1356343	Si	1.5380697	0	-0.7249815	Si	1.1733504	-2.3001762	-0.7980015
Si	1.2743517	-1.3358186	-3.1399453	Si	1.2753634	-1.344217	-2.7316151	Si	1.5677971	-0.4159911	-2.1895272
Si	-1.5434157	0	-1.1356343	Si	-1.5380697	0	-0.7249815	Si	-1.8768885	-0.0947712	-0.6231421
Si	3.7086121	0	-0.1981656	C	-5.0755216	0	-2.2975266	Si	2.3407775	-4.3215179	-0.9723541
Si	-3.7086121	0	-0.1981656	C	-5.8903127	-1.2548252	-2.6003642	Si	-4.1983681	-0.2052228	-1.150224
C	2.4237878	-1.9665689	2.416639	C	-5.8903127	1.2548252	-2.6003642	Si	2.3044001	3.3246248	-1.5607163
H	2.4171276	-2.9266684	2.9435383	H	-4.2008555	0	-2.9622829	C	4.7673778	-3.1875098	-3.2352091
H	2.5500531	-1.1739079	3.1577716	C	-6.378622	-1.2601555	-4.0487166	H	5.800384	-2.8753199	-3.4207675
H	1.4524988	-1.8301103	1.9340826	H	-6.7629604	-1.3009855	-1.9359048	H	4.5288542	-3.9945282	-3.9323332
C	2.4237878	1.9665689	2.416639	H	-5.3010975	-2.1574519	-2.4046945	H	4.110819	-2.3405517	-3.4508895
H	2.5500531	1.1739079	3.1577716	C	-6.378622	1.2601555	-4.0487166	C	1.183006	-4.431221	-4.287625
H	2.4171276	2.9266684	2.9435383	H	-6.7629604	1.3009855	-1.9359048	H	2.1522569	-4.0556817	-4.6224229
H	1.4524988	1.8301103	1.9340826	H	-5.3010975	2.1574519	-2.4046945	H	0.7417891	-5.0144234	-5.1027541
C	-2.4237878	1.9665689	2.416639	C	-7.1736172	0	-4.372602	H	0.5322742	-3.5748091	-4.0915075
H	-2.4171276	2.9266684	2.9435383	H	-6.9863387	-2.1515343	-4.2407083	C	-3.397398	-0.6809466	-4.6540158
H	-2.5500531	1.1739079	3.1577716	H	-5.5086598	-1.3209321	-4.7153339	H	-3.7576653	-1.1194529	-5.5914071
H	-1.4524988	1.8301103	1.9340826	H	-6.9863387	2.1515343	-4.2407083	H	-3.4042183	0.4059735	-4.7585025
C	-2.4237878	-1.9665689	2.416639	H	-5.5086598	1.3209321	-4.7153339	H	-2.3658564	-0.9988614	-4.4953278
H	-2.5500531	-1.1739079	3.1577716	H	-7.4743404	0	-5.4256289	C	-4.2738515	2.7999208	-2.9522751
H	-2.4171276	-2.9266684	2.9435383	H	-8.0998357	0	-3.7827334	H	-4.7024052	2.3108281	-3.8305392
H	-1.4524988	-1.8301103	1.9340826	C	5.0755216	0	-2.2975266	H	-4.5398524	3.8612686	-2.9919859
C	-3.6263509	-3.487787	0.0683775	C	5.8903127	1.2548252	-2.6003642	H	-3.1857386	2.7165578	-3.0155847
H	-4.4738061	-3.6054391	-0.6110065	C	5.8903127	-1.2548252	-2.6003642	C	0.0355155	4.5506996	-3.9724275
H	-2.7141388	-3.4292675	-0.5319469	H	4.2008555	0	-2.9622829	H	-0.74857	5.2725628	-4.224242
H	-3.5704276	-4.3797596	0.701547	C	6.378622	1.2601555	-4.0487166	H	0.8078778	4.609611	-4.7432741
C	-5.4531005	-2.0802038	2.0601931	H	6.7629604	1.3009855	-1.9359048	H	-0.3991971	3.5481619	-3.9967306
H	-6.2960268	-2.1309331	1.366389	H	5.3010975	2.1574519	-2.4046945	C	2.7742571	1.9396216	-4.8559195
H	-5.4626558	-2.9899159	2.6700776	C	6.378622	-1.2601555	-4.0487166	H	1.9538906	2.565197	-5.2139166
H	-5.613293	-1.2277826	2.7250504	H	6.7629604	-1.3009855	-1.9359048	H	3.4863453	1.8068717	-5.6779797
C	-5.4531005	2.0802038	2.0601931	H	5.3010975	-2.1574519	-2.4046945	H	2.3692786	0.9628074	-4.5869849
H	-5.613293	1.2277826	2.7250504	C	7.1736172	0	-4.372602	C	4.4022019	4.3474203	-4.0998782
H	-5.4626558	2.9899159	2.6700776	H	6.9863387	2.1515343	-4.2407083	H	4.9550427	4.9177437	-3.3523039
H	-6.2960268	2.1309331	1.366389	H	5.5086598	1.3209321	-4.7153339	H	5.0895242	4.1000952	-4.916146
C	-3.6263509	3.487787	0.0683775	H	6.9863387	-2.1515343	-4.2407083	H	3.618967	4.9905548	-4.5095504
H	-2.7141388	3.4292675	-0.5319469	H	5.5086598	-1.3209321	-4.7153339	C	5.0880793	1.6270116	-2.8682209
H	-4.4738061	3.6054391	-0.6110065	H	7.4743404	0	-5.4256289	H	5.7118822	2.1101744	-2.111971
H	-3.5704276	4.3797596	0.701547	H	8.0998357	0	-3.7827334	H	4.7058681	0.6920461	-2.451259
C	3.6263509	-3.487787	0.0683775	C	-4.4732429	1.8528295	0.7841204	H	5.7225505	1.3832961	-3.7266335
H	2.7141388	-3.4292675	-0.5319469	C	-5.930465	2.2257651	1.0526443	C	1.5584602	6.6434934	-2.3762868
H	4.4738061	-3.6054391	-0.6110065	C	-3.6669867	1.8916967	2.0822177	H	2.4062417	6.6328057	-3.0654906
H	3.5704276	-4.3797596	0.701547	H	-4.0519982	2.6107045	0.107916	H	0.8343247	7.3778625	-2.7447099
C	5.4531005	-2.0802038	2.0601931	C	-6.036144	3.5916436	1.7324847	H	1.9172512	6.9858097	-1.4030667

H	5.4626558	-2.9899159	2.6700776
H	6.2960268	-2.1309331	1.366389
H	5.613293	-1.2277826	2.7250504
C	5.4531005	2.0802038	2.0601931
H	6.2960268	2.1309331	1.366389
H	5.4626558	2.9899159	2.6700776
H	5.613293	1.2277826	2.7250504
C	3.6263509	3.487787	0.0683775
H	4.4738061	3.6054391	-0.6110065
H	2.7141388	3.4292675	-0.5319469
H	3.5704276	4.3797596	0.701547
Si	-3.8100533	-1.9511219	1.1415814
Si	-3.8100533	1.9511219	1.1415814
Si	3.8100533	1.9511219	1.1415814
Si	3.8100533	-1.9511219	1.1415814
Si	-5.4744897	0	-1.7733414
Si	5.4744897	0	-1.7733414
C	-7.1542498	0	-0.8869672
H	-7.7335085	0.8840649	-1.1683378
H	-7.7335085	-0.8840649	-1.1683378
H	-7.0558143	0	0.2001049
C	7.1542498	0	-0.8869672
H	7.7335085	-0.8840649	-1.1683378
H	7.7335085	0.8840649	-1.1683378
H	7.0558143	0	0.2001049
C	-5.3926925	-1.5376598	-2.8629191
H	-5.6042259	-2.4376295	-2.2804984
H	-6.1389061	-1.4662583	-3.6616264
H	-4.4024784	-1.6494906	-3.3130478
C	-5.3926925	1.5376598	-2.8629191
H	-6.1389061	1.4662583	-3.6616264
H	-5.6042259	2.4376295	-2.2804984
H	-4.4024784	1.6494906	-3.3130478
C	5.3926925	1.5376598	-2.8629191
H	5.6042259	2.4376295	-2.2804984
H	6.1389061	1.4662583	-3.6616264
H	4.4024784	1.6494906	-3.3130478
C	5.3926925	-1.5376598	-2.8629191
H	6.1389061	-1.4662583	-3.6616264
H	5.6042259	-2.4376295	-2.2804984
H	4.4024784	-1.6494906	-3.3130478
H	-6.3916427	1.4766218	1.7080342
H	-6.5120522	2.2298969	0.1264404
C	-3.7625999	3.2557437	2.7615302
H	-4.0503566	1.1265048	2.7710814
H	-2.6189885	1.6344938	1.8897541
C	-5.2147019	3.644373	3.0155615
H	-7.0850574	3.8272806	1.944338
H	-5.6737124	4.361916	1.0394857
H	-3.1997287	3.252161	3.7016502
H	-3.2936704	4.0088996	2.1151344
H	-5.2709811	4.6426676	3.4620784
H	-5.6477015	2.9492764	3.7469253
C	-4.4732429	-1.8528295	0.7841204
C	-3.6669867	-1.8916967	2.0822177
C	-5.930465	-2.2257651	1.0526443
H	-4.0519982	-2.6107045	0.107916
C	-3.7625999	-3.2557437	2.7615302
H	-4.0503566	-1.1265048	2.7710814
H	-2.6189885	-1.6344938	1.8897541
C	-6.036144	-3.5916436	1.7324847
H	-6.3916427	-1.4766218	1.7080342
H	-6.5120522	-2.2298969	0.1264404
C	-5.2147019	-3.644373	3.0155615
H	-3.1997287	-3.252161	3.7016502
H	-3.2936704	-4.0088996	2.1151344
H	-7.0850574	-3.8272806	1.944338
H	-5.6737124	-4.361916	1.0394857
H	-5.2709811	-4.6426676	3.4620784
H	-5.6477015	-2.9492764	3.7469253
C	4.4732429	1.8528295	0.7841204
C	3.6669867	1.8916967	2.0822177
C	5.930465	2.2257651	1.0526443
H	4.0519982	2.6107045	0.107916
C	3.7625999	3.2557437	2.7615302
H	4.0503566	1.1265048	2.7710814
H	2.6189885	1.6344938	1.8897541
C	6.036144	3.5916436	1.7324847
H	6.3916427	1.4766218	1.7080342
H	6.5120522	2.2298969	0.1264404
C	5.2147019	3.644373	3.0155615
H	3.1997287	3.252161	3.7016502
H	3.2936704	4.0088996	2.1151344
H	7.0850574	3.8272806	1.944338
H	5.6737124	4.361916	1.0394857
H	5.2709811	4.6426676	3.4620784
H	5.6477015	2.9492764	3.7469253
C	4.4732429	-1.8528295	0.7841204
C	5.930465	-2.2257651	1.0526443
C	3.6669867	-1.8916967	2.0822177
H	4.0519982	-2.6107045	0.107916
C	-0.7048758	5.0605775	-1.0582197
H	-1.2215759	4.1003589	-0.982593
H	-0.3659926	5.3448925	-0.0597628
H	-1.428512	5.8075995	-1.4000336
C	-4.3244096	3.0883498	0.0864603
H	-4.797256	2.776435	1.0200974
H	-3.2418648	3.0168355	0.213357
H	-4.5767308	4.1396071	-0.0854733
C	-6.8081663	2.1247603	-1.377676
H	-7.2336194	1.7612338	-0.4389159
H	-7.1235168	3.1654218	-1.5081526
H	-7.2345398	1.5378448	-2.1939263
C	-6.2816773	-0.8352832	-3.8258888
H	-6.3746004	0.2264952	-4.0676069
H	-6.501673	-1.4036305	-4.7361831
H	-7.0390402	-1.0834008	-3.0807693
C	-4.3908948	-3.1274886	-3.1161994
H	-3.4010871	-3.4193318	-2.7570315
H	-5.1400549	-3.5343427	-2.4328746
H	-4.547083	-3.583414	-4.0995369
C	5.1844698	-2.3555289	-0.3257038
H	4.6104132	-1.4397875	-0.4884078
H	5.096367	-2.621706	0.7299551
H	6.2369557	-2.1405628	-0.5385939
C	5.683622	-5.2514195	-1.1698584
H	6.7147167	-5.0114309	-1.4496447
H	5.6831463	-5.5572138	-0.1206084
H	5.3595667	-6.1032705	-1.7728744
C	2.428941	-6.9955837	-3.1799968
H	2.5656089	-7.6545647	-2.3192099
H	1.9613618	-7.577197	-3.981271
H	3.4165819	-6.6802951	-3.5264035
C	-0.3661097	-6.123556	-2.2684133
H	-0.3171126	-6.8340059	-1.4397137
H	-1.0076119	-5.2905989	-1.9691276
H	-0.8365077	-6.6272954	-3.1191211
Si	3.6748566	4.2342418	0.1443873
Si	2.1346847	-5.4381719	1.1092308
Si	-5.5243186	-1.3951195	0.4160382
Si	0.7266521	4.9554009	-2.270263
Si	3.6784033	2.7441466	-3.4173351
Si	4.5933021	-3.7420477	-1.4473988
Si	1.3431562	-5.5160052	-2.761315
Si	-4.5284007	-1.2571159	-3.2678117
Si	-4.9258576	2.046341	-1.3565056
C	-7.2151601	-1.7896795	-0.3164008
H	-7.1464883	-2.4760224	-1.1631361
H	-7.8229698	-2.2720864	0.456816
H	-7.7413717	-0.8899651	-0.6436326
C	5.0092565	5.3590313	-0.5627952

C	6.036144	-3.5916436	1.7324847
H	6.3916427	-1.4766218	1.7080342
H	6.5120522	-2.2298969	0.1264404
C	3.7625999	-3.2557437	2.7615302
H	4.0503566	-1.1265048	2.7710814
H	2.6189885	-1.6344938	1.8897541
C	5.2147019	-3.644373	3.0155615
H	7.0850574	-3.8272806	1.944338
H	5.6737124	-4.361916	1.0394857
H	3.1997287	-3.252161	3.7016502
H	3.2936704	-4.0088996	2.1151344
H	5.2709811	-4.6426676	3.4620784
H	5.6477015	-2.9492764	3.7469253
Sn	-4.1029826	0	-0.3312075
Sn	4.1029826	0	-0.3312075

H	5.5493627	5.8231413	0.2695066
H	5.7340032	4.8035042	-1.1622633
H	4.5907202	6.157782	-1.179276
C	2.8361145	-7.1772407	0.9464163
H	3.8770575	-7.1691396	0.6152735
H	2.7932555	-7.6784558	1.9190389
H	2.2576096	-7.7735703	0.2362082
C	2.6194958	5.2915913	1.2905116
H	3.1997038	5.6053034	2.1636933
H	2.2878691	6.1918964	0.7668222
H	1.7306269	4.7654612	1.6424511
C	4.5396315	2.8488817	1.0752522
H	5.2475884	2.3420031	0.4138778
H	5.0964754	3.2478693	1.9288077
H	3.8365619	2.0977927	1.4401188
C	3.101169	-4.5024674	2.4227974
H	2.9531179	-4.9751821	3.3990763
H	4.1722339	-4.5040468	2.2051477
H	2.771557	-3.4626572	2.4936687
C	0.337303	-5.5698689	1.6395258
H	-0.2530831	-6.1371618	0.9163555
H	0.2810819	-6.0844591	2.6044887
H	-0.1205993	-4.5836041	1.7501894
C	-4.707908	-3.029848	0.8592523
H	-5.2561732	-3.5144415	1.6734404
H	-4.7152834	-3.7012283	-0.0032132
H	-3.6697915	-2.9016364	1.1708697
C	-5.8276727	-0.3432538	1.944413
H	-6.4539113	0.5174562	1.6945797
H	-6.34872	-0.9298906	2.7077922
H	-4.8996181	0.0296424	2.3774706
Si	-0.3478224	1.0223006	3.4490829
Si	-1.2477162	-0.8666518	4.6016049
Si	1.7935345	1.3578834	4.4691071
Si	-1.6914262	2.9136182	3.9657574
C	2.4299373	3.1003855	4.1847355
H	2.6277931	3.2965292	3.1322672
H	1.7240743	3.8530922	4.5435909
H	3.3692163	3.2298265	4.732621
C	-3.5126727	2.4584524	3.8666625
H	-3.7688971	2.0459759	2.889742
H	-3.7787324	1.720564	4.6274562
H	-4.1278287	3.3492556	4.0296579
C	0.0248111	-2.2481996	4.6960391
H	0.3369006	-2.566653	3.6986914
H	0.9142576	-1.9466126	5.2537265
H	-0.4173957	-3.1107457	5.2053906
C	-2.8291643	-1.5413231	3.8578149
H	-3.6474728	-0.8276071	3.9662854
H	-2.714206	-1.78216	2.8010891

H	-3.1084965	-2.4571282	4.3894056
C	-1.3402274	4.3314113	2.7847043
H	-0.3067943	4.6740339	2.8782811
H	-1.5084992	4.0445407	1.744952
H	-1.9979701	5.1759879	3.0140219
C	3.123943	0.1296989	3.9698685
H	2.7967832	-0.9012396	4.121778
H	3.4179772	0.2319364	2.924681
H	4.0025847	0.306862	4.5999629
C	-1.7045478	-0.3463639	6.3567945
C	1.5843779	1.1604589	6.3332705
C	-1.3515077	3.5183563	5.716822
H	1.3767918	0.126919	6.6172661
H	-1.5391983	2.7423038	6.4620327
H	-2.0733201	-1.2326081	6.8850579
H	-2.0215962	4.3581215	5.9300048
H	0.8010218	1.7982616	6.7458435
H	-0.8677097	0.0604737	6.9242031
H	-2.5087186	0.393507	6.3528259
H	2.5334372	1.4488024	6.7986696
H	-0.3250267	3.8704857	5.8393604

6. References

1. C. Hoch, M. Wendorff and C. Röhr, *J. Alloys Compd.*, 2003, **361**, 206.
2. TURBOMOLE V7.3 2018, a development of University of Karlsruhe and Forschungszentrum Karlsruhe GmbH, 1989-2007, TURBOMOLE GmbH, since 2007.
3. R. Ahlrichs, M. Bär, M. Häser, H. Horn and C. Kölmel, *Chem. Phys. Lett.*, 1989, **162**, 165.
4. J. P. Perdew, K. Burke and M. Ernzerhof, *Phys. Rev. Lett.*, 1996, **77**, 3865.
5. C. Adamo and V. J. Barone, *J. Chem. Phys.*, 1999, **110**, 6158.
6. F. Weigend and R. Ahlrichs, *Phys. Chem. Chem. Phys.*, 2005, **7**, 3297
7. K. Eichkorn, O. Treutler, H. Öhm, M. Häser and R. Ahlrichs, *Chem. Phys. Lett.*, 1995, **240**, 283.
8. F. Weigend, *Phys. Chem. Chem. Phys.*, 2006, **8**, 1057.
9. M. Sierka, A. Hogekamp and R. Ahlrichs, *J. Chem. Phys.* , 2003, **118**, 9136.
10. A. Klamt and G. Schüürmann, *J. Chem. Soc., Perkin Trans. 2*, 1993, 799.
11. D. Rappoport and F. Furche, *J. Chem. Phys.*, 2007, **126**, 201104.
12. F. Furche and R. Ahlrichs, *J. Chem. Phys.*, 2002, **117**, 7433.
13. Jmol: an open-source Java viewer for chemical structures in 3D. http://www.jmol.org/.
14. G. Knizia, *J. Chem. Theory. Comput.*, 2013, **9**, 4834.
